# Antiseptic quaternary ammonium compound tolerance by gram-negative bacteria can be rapidly detected using an impermeant fluorescent dye-based assay

**DOI:** 10.1038/s41598-020-77446-8

**Published:** 2020-11-25

**Authors:** Branden S. J. Gregorchuk, Shelby L. Reimer, Daniel R. Beniac, Shannon L. Hiebert, Timothy F. Booth, Michelle Wuzinski, Brielle E. Funk, Kieran A. Milner, Nicola H. Cartwright, Ali N. Doucet, Michael R. Mulvey, Mazdak Khajehpour, George G. Zhanel, Denice C. Bay

**Affiliations:** 1 Department of Medical Microbiology and Infectious Diseases, University of Manitoba, Rm 514C Basic Medical Sciences Bldg., 745 Bannatyne Avenue, Winnipeg, MB R3E 0J9 Canada; 2 National Microbiology Laboratory, Public Health Agency of Canada, Winnipeg, MB Canada; 3 Department of Chemistry, University of Manitoba, Winnipeg, MB Canada

**Keywords:** Biochemistry, Biological techniques, Microbiology, Environmental sciences, Medical research

## Abstract

Biocides such as quaternary ammonium compounds (QACs) are potentially important contributors towards bacterial antimicrobial resistance development, however, their contributions are unclear due to a lack of internationally recognized biocide testing standards. Methods to detect QAC tolerance are limited to laborious traditional antimicrobial susceptibility testing (AST) methods. Here, we developed a rapid fluorescent dye-based membrane impermeant assay (RFDMIA) to discriminate QAC susceptibility among Gram-negative Enterobacterales and Pseudomonadales species. RFDMIA uses a membrane impermeant fluorescent dye, propidium iodide, in a 30-min 96-well fluorescent microplate-based assay where cell suspensions are exposed to increasing QAC concentrations. Our results demonstrate that RFDMIA can discriminate between QAC-susceptible and QAC-adapted *Escherichia coli* tolerant phenotypes and predict benzalkonium and cetrimide tolerance in all species tested except for intrinsically fluorescent *Pseudomonas aeruginosa*. RFDMIA identified a close association to minimum inhibitory concentration values determined by broth microdilution AST and increasing fluorescent dye emission values. RFDMIA emission values and scanning electron microscopy results also suggest that CET-adapted *E. coli* isolates have a CET dependence, where cells require sub-inhibitory CET concentrations to maintain bacilliform cell integrity. Overall, this study generates a new, rapid, sensitive fluorescent assay capable of detecting QAC-susceptible Gram-negative bacteria phenotypes and cell membrane perturbations.

## Introduction

Quaternary ammonium compounds encompass a diverse group of positively-charged, nitrogen-containing molecules used as antiseptics/disinfectants, but also as industrial surfactants and lipophilic intercalating dyes. QAC antiseptics/disinfectants are heavily relied upon by clinical facilities^1,2^ and various industries^3–5^, and are pervasive in daily-use commercially available items such as household cleaners, cosmetics, and oral hygiene products^6–8^. Benzalkonium chloride (BZK) is one of the most frequently overused QACs in household and industrial cleaners^8,9^ and is formulated as a mixture of alkyl chains (C_12_ and C_14_) linked to a benzyl-dimethyl-ammonium chloride moiety^4,10–12^. Cetrimide (CET) is another widely used QAC added to antiseptic creams, optical and oral hygiene solutions, as well as numerous cleansers^11,13^. CET is formulated as a mixture of *n*-alkyl chains (C_8_ to C_18_) linked to a trimethyl ammonium bromide moiety^11^.

CET and BZK represent some of the most popular antiseptics in use and when they are used at recommended working concentrations (0.001 to 0.01% w/v). Within minutes, they can rapidly disinfect surfaces that are contaminated with bacteria. QAC compounds act by displacing bacterial phospholipids, enabling them to enter the cell and denature proteins and/or increase reactive oxygen/nitrogen species formation^11,14,15^. QAC mechanisms of action result in a generalized loss of bacterial membrane fluidity, solubilization of membrane lipids and proteins, and cell leakage, culminating in bacterial death^11,16,17^. Previous studies have shown that many proteobacterial species considered to be critical priority antimicrobial resistant (AMR) pathogens (e.g. Enterobacteriales, *Acinetobacter* spp., and *Pseudomonas* spp.) are intrinsically tolerant to higher concentrations of QACs^18,19^. These species have demonstrated an ability to adapt to QACs upon prolonged or repeated sub-lethal concentration exposures, ultimately leading to increased QAC tolerance, biocide cross-tolerance, as well as cross-resistance to clinically relevant antibiotics^20–22^. This is concerning as annual global QAC usage is 100 times greater than that of therapeutic antibiotics^23,24^. QAC overuse has now made these compounds a common pollutant in wastewater and soil environments^4,15,23^. Due to our reliance on and overuse of QACs in products, it is essential to understand how QACs may be drivers of antimicrobial resistance.

One of the major knowledge gaps in bacterial antiseptic/disinfectant susceptibility studies is the lack of routine, standardized, clinical antimicrobial susceptibility tests to measure QAC tolerance. QAC testing is not covered by the European Committee on Antimicrobial Susceptibility Testing (EUCAST) or the Clinical and Laboratory Standards Institute (CLSI); QACs lack defined breakpoint concentrations that can be used for antimicrobial susceptibility testing (AST)^25,26^. Consequently, QAC “resistance” values cannot be standardized and are referred to herein as “tolerance” values^27^. Current approaches to measure QAC tolerance values involve traditional AST methods such as agar dilutions or broth microdilution testing of bacterial isolates against various reference strains^27–30^. These laborious and time consuming (requiring 24–48 h) methods are the only established techniques to measure QAC minimum inhibitory concentration (MIC) values and minimum biocidal concentration (MBC) values. Hence, there is a considerable need for a rapid and sensitive assay to determine antiseptic QAC susceptibilities and overcome these current knowledge gaps.

Here, we have developed a rapid (30-min) fluorescent dye-based membrane integrity assay referred to as “RFDMIA” to measure QAC susceptibility. RFDMIA measures the difference in membrane integrity between QAC-tolerant and susceptible bacterial suspensions. RFDMIA uses a membrane impermeant dye, propidium iodide (PI), to indirectly measure changes in bacterial membrane permeability. We hypothesize that, in the presence of increasing concentrations of QACs, but identical PI concentrations, QAC-susceptible bacteria can be distinguished from QAC-tolerant isolates by monitoring differences in fluorescent dye emission (EM). Hence, increased PI penetration and subsequent PI binding to DNA/RNA in QAC-susceptible isolates will cause an increase in the dye’s fluorescent EM that can be monitored as a relative change in fluorescent EM values over a 30-min period (i.e. PI ΔRFU_Δ30min_). This difference in fluorescent dye EM can be used to discriminate between QAC susceptible and tolerant bacteria. Additionally, we hypothesize that QAC-tolerant bacterial cells will possess greater membrane integrity in the presence of QACs, allowing lower PI penetration that will result in reduced PI ΔRFU_Δ30min_ values at concentrations below or at their respective MIC or MBC value.

To test our hypotheses, we compared BZK and CET MIC values using broth microdilution AST methods to PI ΔRFU_Δ30min_ values collected from RFDMIA experiments for *Escherichia coli* K-12 BW25113 isolates adapted to BZK or CET. To confirm that 30-min of QAC exposure time was sufficient for bactericidal activity, a 30-min MBC (30MBC) value was measured by spot plating bacterial cells directly from each RFDMIA plate. The outcome from this analysis demonstrated that the assay was able to discriminate QAC-tolerant from QAC-susceptible *E. coli* and suggested a membrane integrity phenotype that we describe as “QAC-dependent” when we visualized and analyzed these cells using scanning electron microscopy (SEM). This study also compares the use of another impermeant fluorescent dye as a replacement for PI, SYTOX Blue, and revealed that both dyes were sufficiently sensitive for QAC susceptibility prediction for *E. coli* by RFDMIA. To determine assay robustness in estimating QAC tolerance in other proteobacterial species, we also performed RFDMIA with *Acinetobacter baumannii*, *Pseudomonas aeruginosa, Shigella sonnei*, and *Klebsiella pneumoniae* isolates. Comparison of additional species revealed that RFDMIA could successfully discern their QAC susceptibility except for intrinsically fluorescent *Pseudomonas aeruginosa.* Overall, this study generates a new, rapid, sensitive fluorescent assay capable of detecting QAC-susceptible Gram-negative bacteria phenotypes and cell membrane perturbations.

## Results

### RFDMIA can discriminate QAC-susceptible from QAC-tolerant E. coli

To determine the accuracy of the RFDMIA as a technique for rapidly predicting and detecting QAC susceptibility based on PI ΔRFU_Δ30min_ at EM 620 nm, we focused our initial analyses on *E. coli* K12 BW25113 isolates resuspended in phosphate buffered saline (PBS). In addition to an unadapted control strain, BW25113 was adapted to either BZK (ECBZKT) or CET (ECCETT) using a gradual drug exposure experiment^31^. Here, unadapted *E. coli* isolates were grown in the presence of increasing sub-inhibitory concentrations of BZK or CET over 40 successive sub-cultures (as described in BioRxiv #201,814; currently under review). Afterwards, the final MIC of each QAC-adapted *E. coli* isolate was compared to the unadapted parental BW25113 (EC) strain to determine the extent of their QAC tolerance. Both QAC-adapted isolates demonstrated 4- to eightfold higher MIC values to their respective QACs based on corresponding AST data (Table 1) making them useful comparative study models. Our initial RFDMIA analyses sought to distinguish significant differences in PI EM 620 nm values (ΔRFU_Δ30min_) when incubated with stationary phase culture cell preparations exposed to increasing concentrations of BZK or CET over a 30-min timeframe. In the same assays, RFUs corresponding to identical cell sample preparations that were heat-treated with added PI and QACs served as cell membrane disrupted controls to help establish maximum PI RFU_30min_ EM 620 nm values for each QAC concentration tested (Fig. S1). Although differences were observed in PI RFU_30min_ values at EM 620 nm, when we compared live cell suspensions to their identical heat-treated controls, the live cell samples unexposed to QACs (0.0 μg/mL QAC) often had some background RFU_30min_ emission signal between assays. To compensate, we subtracted RFU_30min_ 0.0 μg/mL QAC value as a calculation adjustment, resulting in ΔRFU_Δ30min_ values to observe accurate increases due to QAC addition (Fig. S1).

**Table 1:** A summary of mean BZK and CET MIC and 30MBC values of all bacterial isolates tested in this study based on a twofold (log_2_) dilution series from three bacterial bioreplicates measured in technical triplicate.

Bacterial isolate tested in this study; isolate abbreviation	MIC (μg/mL) (n = 9)*		30MBC (μg/mL) (n = 9)*	
	BZK	CET	BZK	CET
*Escherichia coli* K-12 BW25113; **EC**^a^	18.8	37.5	75.0	75.0
*Escherichia coli* K-12 BW25113; **ECBZKT**^b^	150.0	150.0	150.0	150.0
*Escherichia coli* K-12 BW25113; **ECCETT**^c^	75.0	300.0	150.0	300.0
*Acinetobacter baumannii* DSM 6974; **AB**^d^	18.8	37.5	75.0	75.0
*Pseudomonas aeruginosa* PA01 DSM 22,644; **PA**^d^	75.0	150.0	150.0	300.0
*Klebsiella pneumoniae* DSM 6135; **KP**^d^	18.8	–	37.5	–
*Klebsiella pneumoniae* DSM 6135; **KPBZKT**^e^	75.0	–	150.0	–
*Shigella sonnei* DSM 5570; **SS**^d^	9.4	–	37.5	–
*Shigella sonnei* DSM 5570; **SSBZKT**^f^	37.5	–	75.0	–

*n = 9 refers to 3 biological replicate cultures and 3 technical replicate dilutions used for AST.

^a^Isolate sourced from the Yale Coli Genetic Stock Centre; CGSC (https://cgsc.biology.yale.edu/).

^b^BZK-adapted BW25113 isolate, tolerant to 75 µg/mL BZK (BZKR). Refer to reference BioRXIV# 201,814.

^c^CET-adapted BW25113 isolate, tolerant to 150 µg/mL CET (CETR). Refer to reference BioRXIV# 201,814.

^d^Strain sourced from the Leibniz Institute DSMZ-German Collection of Microorganisms and Cell Cultures GmbH; DSMZ (https://www.dsmz.de/).

^e^BZK-adapted DSM 6135 isolate, tolerant to 75 µg/mL BZK.

^f^BZK-adapted DSM 5570 isolate, tolerant to 37.5 µg/mL BZK.

**Figure 1:**
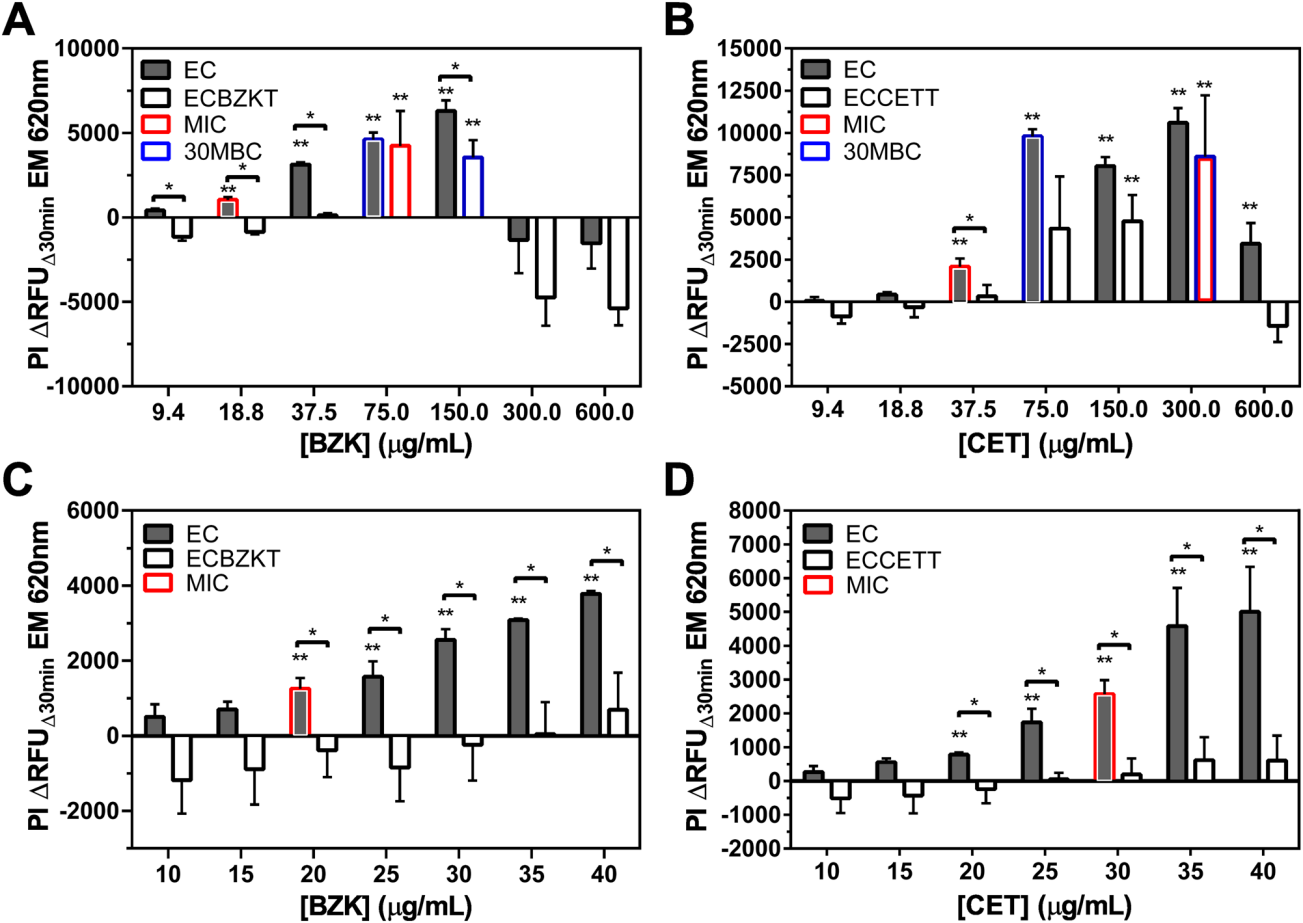
RFDMIA of stationary phase EC, ECBZKT, ECCETT exposed to increasing concentrations of BZK or CET. (**A**) RFDMIA comparison of EC and ECBZKT isolates exposed to BZK. (**B**) Shows RFDMIA results for EC and ECCETT exposed to CET. (**C**) Narrow range RFDMIA of BZK concentrations (10–40 μg/mL). **D**) RFDMIA of EC versus ECCETT exposed to narrow range of CET concentrations (10–40 μg/mL). All graphs are the mean PI _Δ_RFU_Δ30min_ at EM 620 nm values from stationary phase bacterial isolate preparations separately exposed to increasing QAC concentrations. Bar outlines are color coordinated to indicate QAC concentrations corresponding to the MIC (red) and 30MBC (blue) values of each isolate every panel (Table 1). For each RFDMIA plot shown, a Student’s two-tailed *t* test was calculated by comparing every QAC concentration PI ΔRFU_Δ30min_ value to the lowest measured QAC concentration PI ΔRFU_Δ30min_ value shown on the plot. *t* tests were used to identify the lowest QAC concentration in an assay with a significant increase in PI ΔRFU_Δ30min_ value and are indicated as a double asterisk (**) with *P* < 0.01. Data represents three bacterial bioreplicates (n = 3) measured from averaged technical triplicate measurements.

To distinguish QAC-susceptible from tolerant phenotypes among stationary phase *E. coli* cell suspensions using RFDMIA, we sought to identify the lowest QAC concentration where we could detect significantly increased PI ΔRFU_Δ30min_ values for a bacterial isolate as well as QAC concentrations with maximum PI ΔRFU_Δ30min_ values to determine RFDMIA sensitivity and threshold of detection. Based on AST, we exposed cell suspensions to log_2_ increases in QAC concentrations in RFDMIAs; this comparison also allowed us to compare RFDMIA to its respective MIC and 30MBC values determined by AST (Table 1). With this approach, we were able to compare the RFDMIA detection sensitivity and thresholds, but also allowed us to test our hypothesis that PI accessibility should increase as QACs reach a concentration threshold that perturbs cell membrane integrity. At these QAC concentration ranges, PI ΔRFU_Δ30min_ values would be expected to significantly increase at QAC concentrations below or at their respective AST measured MIC value. Figure 1A shows RFDMIA results of EC and ECBZKT isolates, which confirmed that the first significant increase in PI ΔRFU_Δ30min_ values occurred at the lowest BZK concentration tested for both EC (18.8 µg/mL) and ECBZKT (75.0 µg/mL), and both also coincided to their respective MIC values. This outcome shows that for BZK, significant increases in RFDMIA PI ΔRFU_Δ30min_ values correspond to and may predict MIC values for EC and ECBZKT isolates. Additionally, we found that we were able to distinguish between EC and ECBZKT between 9.4 µg/mL to 150 µg/mL BZK in agreement with our hypothesis that the RFDMIA can distinguish between BZK-susceptible and tolerant *E. coli*. Together, the results from this RFDMIA indicates that we were able to distinguish between the tolerant and susceptible isolates using a log_2_ concentration range.

A similar outcome for RFDMIA PI ΔRFU_Δ30min_ values of EC exposed to CET was demonstrated, where both the MIC values and EC RFDMIA occurred at the lowest CET concentration with significantly increased PI ΔRFU_Δ30min_ value (37.5 µg/mL; Fig. 1B). However, RFDMIA of ECCETT isolates demonstrated that the lowest CET concentration with significantly increased PI ΔRFU_Δ30min_ values occurred at 150.0 µg/mL CET, which was twofold lower than its respective CET MIC value for ECCETT (Fig. 1B; Table 1). This indicates that PI dye permeates into ECCETT cells at lower CET concentrations than BZK exposed ECBZKT cells and suggests that ECCETT isolate cell membranes are more permeable to PI dye as noted by the ΔRFU_Δ30min_ value increases. Similar to the results of the BZK RFDMIA, we were able to distinguish between EC and ECCETT at the MIC of EC (37.5 µg/mL; Fig. 1B; Table 1) Overall, PI ΔRFU_Δ30min_ values for both ECBZKT and ECCETT isolates were distinguishable from EC PI ΔRFU_Δ30min_ values at the identical QAC concentrations, validating our main study hypothesis that RFDMIA can distinguish between QAC-susceptible and tolerant phenotypes.

A comparison of RFDMIA PI ΔRFU_Δ30min_ and 30MBC values for EC and QAC-adapted isolates exposed to BZK or CET demonstrated that the 30MBC often coincided to each respective isolate’s maximum PI ΔRFU_Δ30min_ value (Fig. 1A,B). The only exception appeared to be EC exposed to BZK, where its 30MBC value occurred at the second highest PI ΔRFU_Δ30min_ value (75.0 µg/mL BZK) rather than at its maxima (150.0 µg/mL BZK). This finding suggests that RFDMIA maximum PI ΔRFU_Δ30min_ values are not reliable predictors of 30MBC values, nor they are useful measures for discriminating between QAC-susceptible and tolerant phenotypes as maximum PI ΔRFU_Δ30min_ are reached by all cells at these high QAC concentrations.

To determine the precision of the RFDMIA, we tested EC, ECCETT, and ECBZKT isolates against a narrow range of BZK or CET concentrations (0–40 µg/mL in 5 µg/mL steps; Fig. 1C,D). ECBZKT isolates exhibited low but significant gains in PI ΔRFU_Δ30min_ at concentrations of 40 μg/mL BZK, whereas the susceptible EC isolate showed its first significant increase in PI ΔRFU_Δ30min_ value at 20 µg/mL BZK, which corresponded to its respective BZK arithmetic MIC value (Fig. 1C; Table 1). Additionally, from 20 µg/mL BZK to 40 µg/mL BZK, we were able to consistently differentiate between the tolerant and adapted isolates. Similar to our abovementioned results (Fig. 1A,B), our findings suggest that ECBZKT may also exhibit a low level of PI dye permeability in the presence of sub-lethal QAC concentrations. Using the same narrow range of concentrations for CET (0–40 µg/mL; 5 µg/mL steps; Fig. 1D), RFDMIA results for ECCETT and EC indicated that EC had low but significantly increased PI ΔRFU_Δ30min_ values at 20 µg/mL CET, 10 µg/mL lower than it’s expected MIC. Similar to the narrow range BZK RFDMIA results, we were able to distinguish between EC and ECCETT from 20 µg/mL CET to 40 µg/mL CET. Importantly, our results indicate that we were able to use a low QAC concentration to distinguish QAC susceptibility, which shows that RFDMIA is sensitive to QAC induced PI cell permeation in *E. coli*.

### Stationary phase cultures can reliably be used for RFDMIA-based predictions of *E. coli* QAC susceptibility

**Figure 2:**
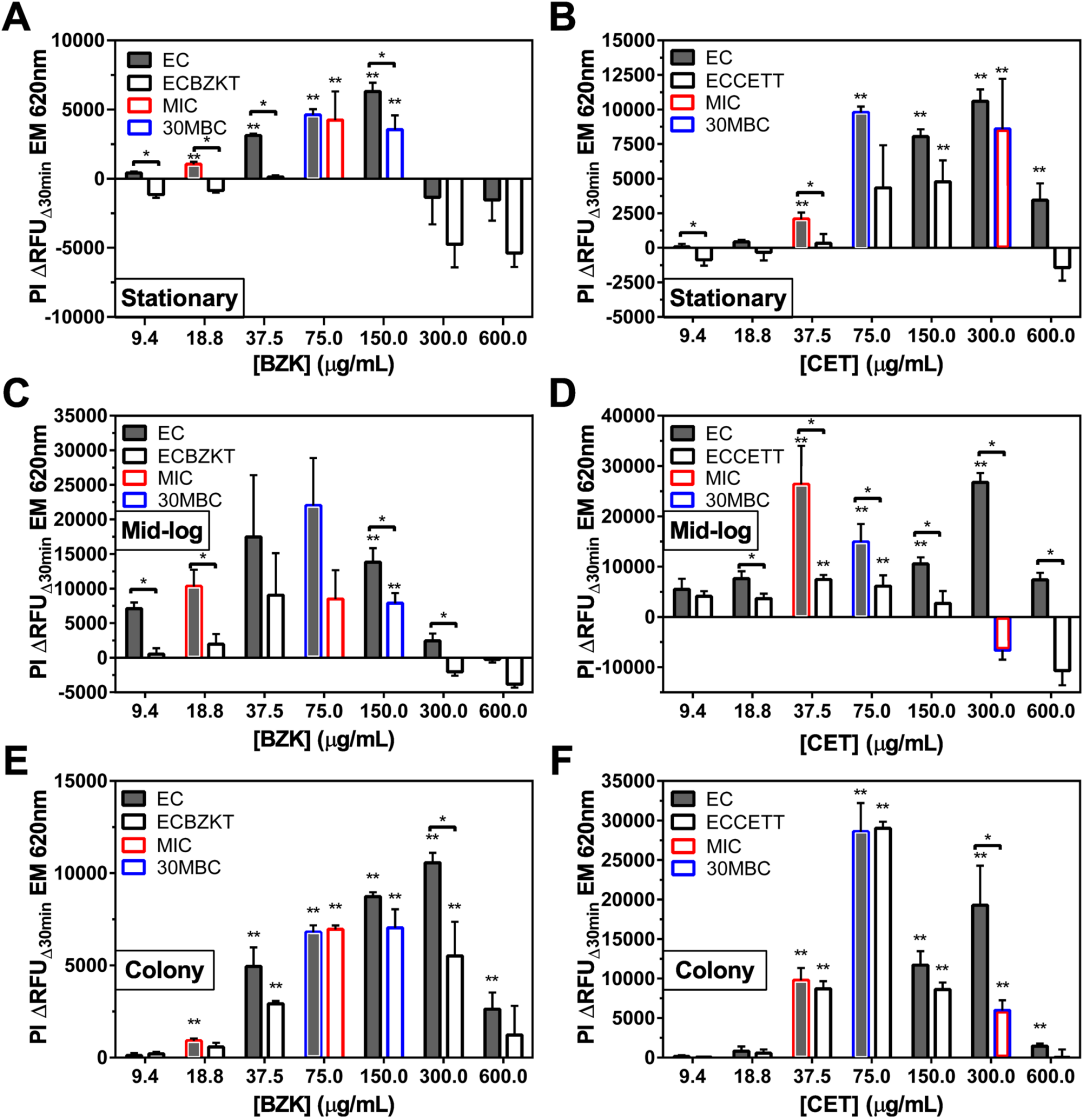
RFDMIA results of EC, ECBZKT, and ECCETT prepared as stationary phase (**A**, **B**), mid-log phase (**C**, **D**), and agar colony (**E**, **F**) PBS-buffered cell suspensions exposed to increasing concentrations of BZK (**A**, **C**, **E**) and CET (**B**, **D**, **F**). All graphs are show the mean PI ΔRFU_Δ30min_ at EM 620 nm values from bacterial cell preparations at increasing QAC concentrations. Bar outlines are color coordinated to indicate the QAC concentration corresponding to the MIC (red) and 30MBC (blue) values of each isolate every panel as determined by AST on Table 1. For each RFDMIA plot shown, a Student’s two-tailed *t* test was calculated by comparing every QAC concentration PI ΔRFU_Δ30min_ value to the lowest measured QAC concentration PI ΔRFU_Δ30min_ value shown on the plot. This *t* test was used to identify the lowest QAC concentration with a significant increase in PI ΔRFU_Δ30min_ value, which is indicated as a double asterisk (**) with *P* < 0.01. Data represents three bacterial bioreplicates (n = 3) measured from averaged technical triplicate measurements.

**Figure 3:**
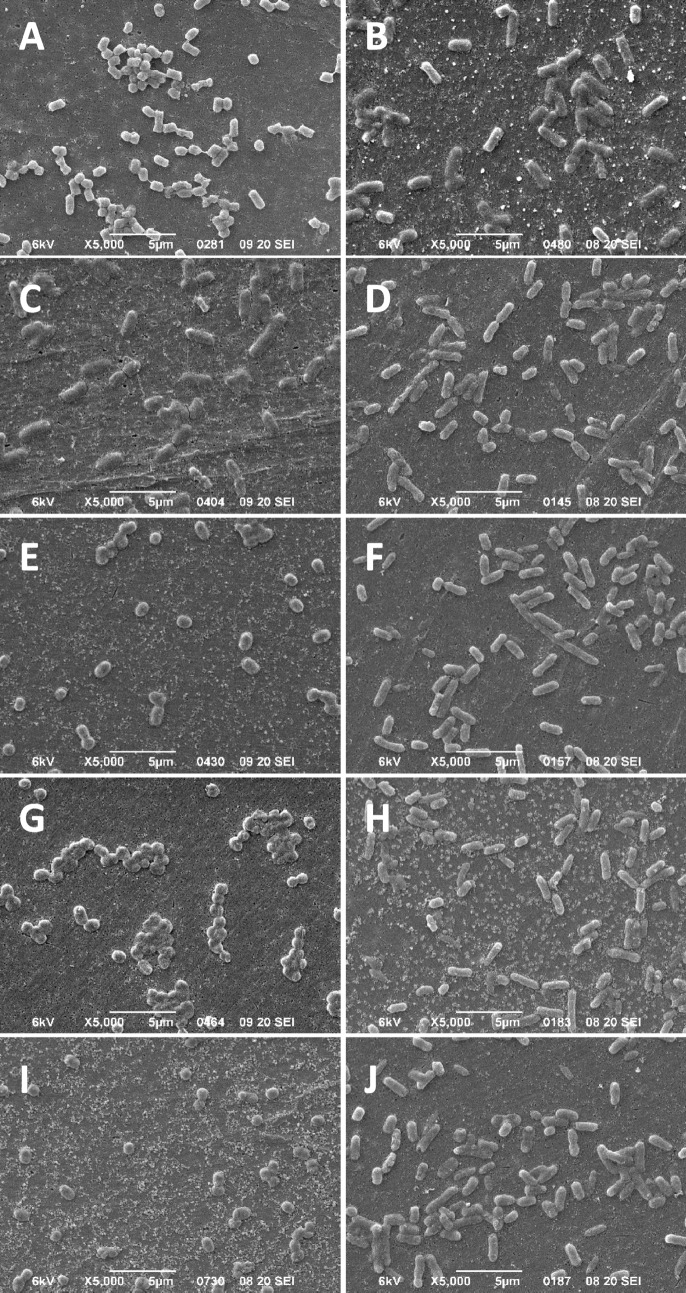
SEM images of EC and ECBZKT exposed to increasing concentrations of BZK for 30-min. (**A**, **C**, **E**, **G**, **I**) show EC isolates and (**B**, **D**, **F**, **H**, **J**) show ECBZKT isolates after 30-min exposure to BZK at 0 µg/mL (**A**, **B**), 9.4 µg/mL (**C**, **D**), 18.8 µg/mL (**E**, **F**), 75 µg/mL (**G**, **H**) and 150 µg/mL (**I**, **J**). All images are representative of five SEM images collected at 5000× magnifications and the white scale bar at the bottom of each panel image indicates 5 µm length.

**Figure 4:**
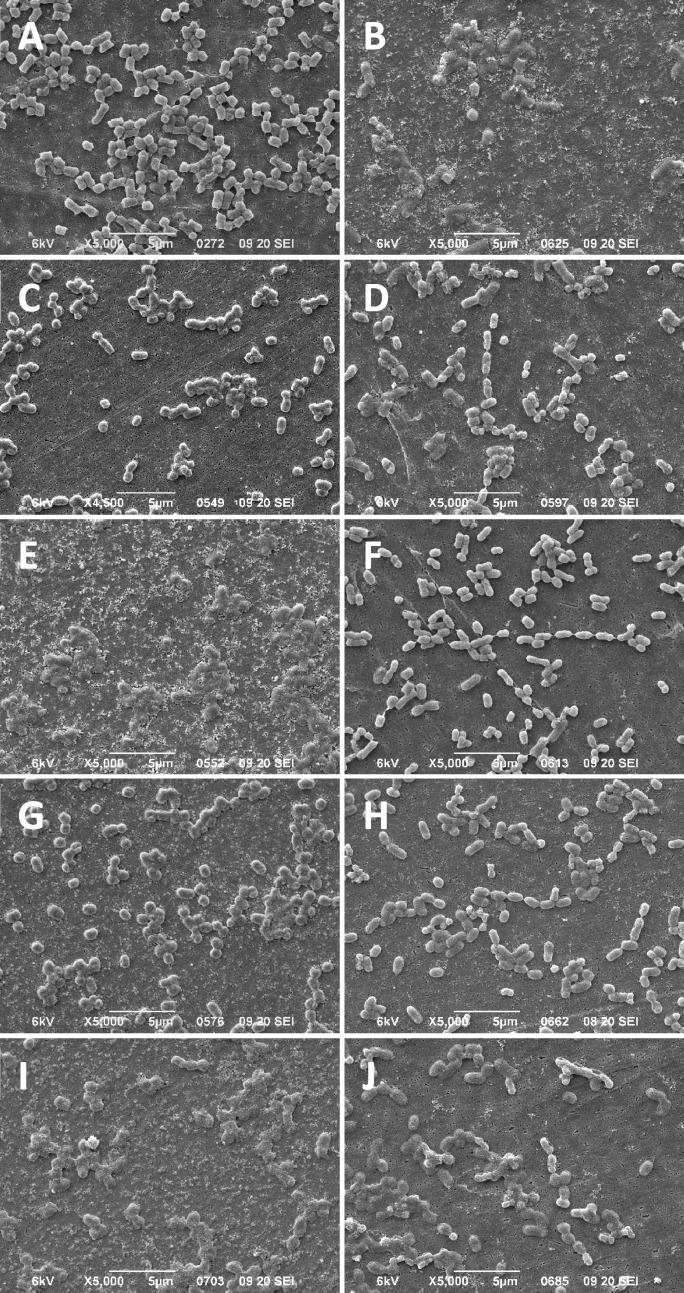
SEM images of EC and ECCETT exposed to increasing concentrations of CET for 30-min. (**A**, **C**, **E**, **G**, **I**) show EC isolates and (**B**, **D**, **F**, **H**, **J**) show ECCETT isolates after 30-min exposure to CET at 0 µg/mL (**A**, **B**), 18.8 µg/mL (**C**, **D**), 37.5 µg/mL (**E**, **F**), 75 µg/mL (G, H) and 300 µg/mL (**I**, **J**). All images are representative of 5 SEM images collected at 5000× magnifications and the white scale bar at the bottom of each panel image indicates 5 µm length.

To further investigate the RFDMIA’s ability to predict QAC susceptibility by different culturing methods frequently used by AST methods, stationary phase cultures initially tested in Fig. 1 were compared to mid-log phase (OD_600nm_ = 0.5) samples and LB agar plate colony suspensions (Fig. 2). After comparing BZK and CET RFDMIA results, it was evident that stationary phase cultures were optimal for efficient RFDMIA for a few important reasons. Firstly, mid-log cells took far longer to prepare, requiring another day’s worth of culturing to collect exponential phase cells. Secondly, mid-log RFDMIA *E.coli* isolates exhibited higher PI ΔRFU_Δ30min_ values for BZK and CET that were 2–threefold higher than stationary phase isolates for each QAC tested with far larger error at increasing QAC concentrations values (Fig. 2C,D). Our attempts to reduce this error by doubling or tripling the number of samples did not improve error. Despite the error, both mid-log ECCETT and ECBZKT RFDMIAs resulted in reproducible differences in PI ΔRFU_Δ30min_ values at QAC concentrations by RFDMIA. ECBZKT and ECCETT mid-log isolate RFDMIAs demonstrated an initial significant increase in PI ΔRFU_Δ30min_ values at QAC concentrations above or below their MIC. For mid-log ECBZKT, the first significant increase in PI ΔRFU_Δ30min_ values occurred at 150.0 μg/mL BZK, twofold higher than its MIC value. Although the first significant increase in PI ΔRFU_Δ30min_ value occurring at 150.0 μg/mL BZK for ECBZKT, it was still possible to distinguish between the tolerant EC and susceptible isolates at sub-inhibitory QAC concentrations 9.4 μg/mL and 18.8 μg/mL BZK (Fig. 2C) by comparing the increase in EM at the unadapted isolates’ MIC value (18.8 μg/mL BZK MIC of EC; Table 1). For mid-log ECCETT, the first significant increase in PI ΔRFU_Δ30min_ occurred at 37.5 μg/mL CET, which was far lower in concentration than ECCETT’s MIC value (Fig. 2D, Table 1). At the CET MIC value of ECCETT (300 μg/mL; Table 1) we did not detect any significant increase in PI ΔRFU_Δ30min_ but similar to ECBZKT, we could distinguish susceptible from tolerant isolates when comparing PI ΔRFU_Δ30min_ value increases (Fig. 2D). For mid-log EC exposed to CET, the first significant increase in PI ΔRFU_Δ30min_ values occurred at 37.5 μg/mL CET, which was in agreement with its MIC value (Fig. 2D; Table 1). Together, these findings revealed that using mid-log cell preparations for RFDMIA had larger error as compared to stationary phase samples and were less reliable for predicting QAC susceptibility at known MIC concentrations. This is potentially due to cell morphology alterations we observed for these adapted isolates based on SEM data (Figs. 3, 4) and known slower growth phenotypes we observed in a recent study involving these isolates (under review; bioRxiv #201814).

EC, ECBZKT, and ECCETT prepared directly from agar plated colonies for RFDMIA demonstrated the least agreement with AST MIC and 30MBC values (Fig. 2E,F). Comparisons of EC to either ECBZKT or ECCETT using colony cell preparations demonstrated PI ΔRFU_Δ30min_ values that were nearly identical in value at all QAC concentrations tested, preventing accurate differentiation of susceptible QAC phenotypes as well as when compared to their respective MIC values (Fig. 2E,F; Table 1). However, EC colony preparations for RFDMIA exposed to BZK or CET correctly predicted MIC as shown by the first significant increase in PI ΔRFU_Δ30min_ value at 18.8 μg/mL and 37.5 μg/mL respectively. Additionally, EC RFDMIA involving BZK showed the assay was unable to predict its 30MBC based on PI ΔRFU_Δ30min_ values, but the assay correctly predicted EC’s CET 30MBC value at 75 μg/mL CET (Fig. 2E,F). Taken together, the fastest and most accurate cell preparation method for RFDMIA involved stationary phase *E. coli* cultures.

### QAC-adapted *E. coli* isolates demonstrate altered cell morphology that may suggest a CET dependent phenotype at sub-inhibitory QAC concentrations

As shown in Fig. 1, ECBZKT and ECCETT RFDMIA unexpectedly resulted in one or more significantly negative ΔRFU_Δ30min_ values at lower QAC concentration ranges (9.4–37.5 μg/mL). QAC-adapted *E. coli* isolates demonstrating negative PI ΔRFU_Δ30min_ values indicate that at these specific QAC concentrations, PI dye permeation was higher in QAC exposed cells than in the same cells lacking any added antimicrobial (0 μg/mL QAC; Fig. 1A,B). Since this was only observed by QAC-adapted *E. coli*, it suggests that their cell integrity may be more compromised and permeant to PI dye than the unadapted EC strain. In an effort to explain what was visually occurring to cells at 0 to 37.5 μg/mL QAC after 30-min of exposure with respect to their cell morphology and cell integrity, we visualized stationary phase EC, ECBZKT, and ECCETT by SEM (Figs. 3, 4, S3, S4; Table S1-S4). To ensure that potential artefacts caused by SEM fixation did not influence our cell morphology interpretations, all image analyses were based on multiple SEM images where 100 cells were counted per QAC concentration, images were blinded for isolate type and QAC concentration, and assessed by two independent assessors to statistically enhance this qualitative analysis.

SEM images of EC without BZK exposure had the classic bacilliform appearance with their mean length being 1.363 ± 0.293 µm and their mean width being 0.742 ± 0.083 µm (Table S1). Stationary phase EC cells lacking any QAC had a predominately inflated appearance (74.5%) indicating that the remaining cells were either intermediate or deflated in appearance (Table S2). When exposed to BZK concentrations below or at its MIC value (18.8 μg/mL BZK), EC took on an increasingly “deflated” appearance (31.54–82.00% deflated) but maintained similar cell length (1.313 ± 0.280 µm) and becme significantly wider (0.921 ± 0.095 µm; *P* < 0.01) than EC cells lacking BZK exposure (Fig. 3A,C; Tables S1, S2). As the concentration of BZK exceeded its MIC value, the EC cells also became shorter and began fusing together (Fig. 3E,G,I). In contrast, SEM analysis of ECBZKT lacking BZK addition had a lower proportion of inflated (46.5%) cells when compared to SEM images of EC cells lacking BZK addition (Fig. 3B; Table S2). At BZK concentrations below ECBZKT’s MIC value (9.4–37.5 μg/mL), the overall appearance of these cells were largely unchanged in inflated abundance (40.5%-46.5%) but, the number of deflated cells decreased (18.5–27.0%) (Table S2). Additionally, ECBZKT also demonstrated elongated cell morphology, when compared to the EC cells with an increased mean length of 0.204 µm (Figs. 3D–F; S1A,B; Table S1). As BZK concentrations reached or exceeded the MIC value of ECBZKT, the cells appeared to be more fused together and deflated (Fig. 3H,I; Table S2). These findings indicate that at low to no BZK concentrations, ECBZKT cell morphology was significantly altered when compared to EC, as determined by a Student’s *t* test (*P* < 0.01) with respect to the adapted isolate’s length and width. Taken together with RFDMIA’s negative PI ΔRFU_Δ30min_ values (Fig. 1), SEM analyses confirm that BZK-adapted *E. coli* cell morphology differs from unadapted EC. These cell morphology differences of QAC-adapted isolates likely play an important role in PI dye/ QAC permeability by comparison to its unadapted cells as we observed by RFDMIA.

Similar trends were observed for *E. coli* SEM CET exposure, where SEM images of EC cells lacking CET exposure showed the classic bacilliform morphology. Upon increasing CET addition, EC cells gradually deflated and dissolved in their appearance as its respective CET MIC value was reached or exceeded (Fig. 4A,C,E,G,I; Tables S3, S4). In contrast, without CET addition ECCETT cells exhibited a greater proportion of deflated cells (68.28% ± 17.99% deflated) when compared to either EC or BZK-adapted isolates when no QAC was present (Fig. 4B; Table S4). ECCETT cells gradually regained an inflated bacilliform morphology (46.5–77.0% inflated) as sub-inhibitory MIC concentrations of CET were added (Fig. 4D,F,H; Table S4). When CET concentrations reached the ECCETT MIC value, cell deflation proportions significantly increased once again (36.50% ± 6.36%; Fig. 4J; Table S4). Similar to ECBZKT isolates, SEM images of ECCETT cells were significantly longer (0.175 μm; *P* = 0.044) and thinner (− 0.081 μm) than EC at all concentrations (Fig. 4; Fig. S4C,D). Additionally, at sub-inhibitory CET MIC values, ECCETT cells began to take on a “chain-like” appearance where they became attached at their poles (Fig. 4D,F). Altogether, RFDMIA analysis demonstrated sufficient sensitivity to predict QAC-adapted isolate cell membrane integrity differences in the *E. coli* isolates and which was confirmed by SEM analysis. RFDMIA and SEM analyses also demonstrated that QAC-adapted *E. coli* have altered cell morphologies, with ECCETT being potentially dependent on the presence of low concentrations of CET for improved cell integrity by SEM imaging.

### High QAC concentrations limit RFDMIA detection accuracy

Another aspect of RFDMIA we observed was the noticeable reduction of RFU_30min_ values at high QAC concentrations (Figs. S1, S2). *E. coli* K-12 isolates and other species we included in this study exhibited a noticeable reduction in RFDMIA PI RFU_30min_ signal at high QAC concentrations (≥ 150 μg/mL) (Fig. S1). Decreased PI RFU_30min_ values at high QAC concentrations corresponded to a noticeable increase in RFU_30min_ EM by live cell preparations and a concomitant decrease in RFU_30min_ EM values of heat-treated cell preparations (Fig. S1). Additionally, the titration of increasing QAC concentrations in the presence of 2.0 μg/mL PI and extracted EC DNA (from OD_600nm_ = 0.2 cells) caused a small, consistent, and significant reduction in PI RFU_30min_ values (8–11% loss in PI RFU_30min_) at every QAC concentration we tested when compared to 0 μg/mL QAC with the exception of BZK at 600 μg/mL (Fig. S2E,F). This finding indicates that without the cell membrane, PI access to DNA in the presence of increasing QACs has a minor and highly consistent quenching effect on PI fluorescence. Hence, the presence of cell membranes in live and heat-treated preparations is an important contributor being ascertained by RFDMIA. When interpreting PI dye EM signal loss at high QAC concentrations in live and heat-treated cell preparations, these reductions are likely due to QAC-membrane interactions. The reduced RFU_30min_ signal values and their convergence by live and heat-treated cell suspensions prohibited our ability to accurately estimate QAC susceptibility at BZK ≥ 150 μg/mL and CET ≥ 300 μg/mL, respectively (Fig. S1). These findings indicate that this fluorescent technique has a maximum QAC concentration detection limit for cell suspensions. It is noteworthy that RFDMIA PI quenching at high QAC concentrations coincided with published critical micelle concentration values of both QACs (CET^32,33^; BZK^34,35^), suggesting that micelle formation by the QAC detergents and cell lipids impedes RFDMIA detection at high QACs.

### RFDMIA can discriminate the QAC susceptibility of *A. baumannii* but not *P. aeruginosa*

**Figure 5:**
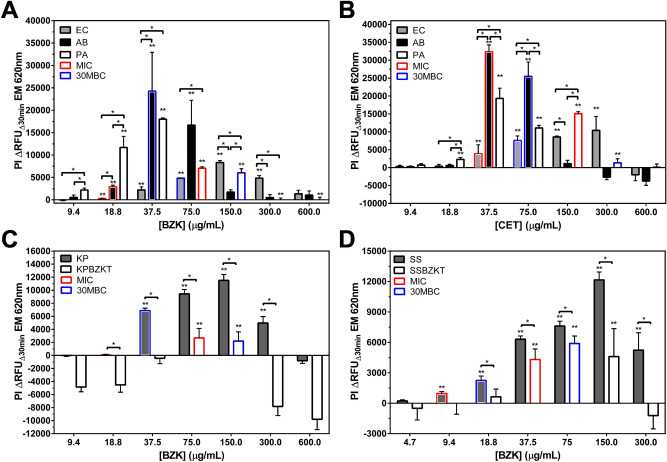
RFDMIA of various Gram-negative species to determine QAC susceptibility. The results of stationary phase RFDMIA of *E. coli* (EC) (**A**, **B**), *A. baumannii* (AB) (**A**, **B**), *P. aeruginosa* (PA) (**A**, **B**)*, K. pneumoniae* (KP; KPBZKT) (**C**), and *S. sonnei* (SS; SSBZKT) (**D**) exposed to increasing concentrations of QAC are shown. For each RFDMIA plot shown, a Student’s two-tailed *t* test was calculated by comparing every QAC concentration PI ΔRFU_Δ30min_ value to the lowest measured QAC concentration PI ΔRFU_Δ30min_ value shown on the plot. This *t* test was used to identify the lowest QAC concentration with a significant increase in PI ΔRFU_Δ30min_ value, which are indicated as a double asterisk (**) with *P* < 0.01. The horizontal lines with asterisks indicate interspecies PI ΔRFU_Δ30min_ value comparisons at the same QAC concentrations. Data represents three bacterial bioreplicates (n = 3) measured from averaged technical triplicate measurements.

To determine how reliable and accurate RFDMIA is at detecting QAC susceptibility phenotypes of other Gram-negative bacterial species, RFDMIA was repeated comparing *E. coli* K-12 BW25113 (EC) to *A. baumannii* (AB) and *P. aeruginosa* (PA) cell suspensions (Fig. 5A,B). RFDMIA results for AB exposed to BZK demonstrated that the first statistically significant increase in PI ΔRFU_Δ30min_ value occurred at 18.8 µg/mL BZK, in agreement with its respective MIC value. Similarly, EC measured in the same RFDMIA experiment as a control reconfirmed that the first significantly increased PI ΔRFU_Δ30min_ value occurred at the same BZK concentration of its respective MIC value (Fig. 5A; Table 1). When exposed to increasing concentrations of CET, both AB and EC had a significant increase in PI ΔRFU_Δ30min_ values at 37.5 µg/mL CET, their respective MICs for both bacteria (Fig. 5B; Table 1), indicating that RFDMIA can accurately discriminate the QAC susceptibility of *A. baumannii* as we observed for *E. coli*.

AST MIC values of *P. aeruginosa* revealed that this species has greater intrinsic tolerance to BZK and CET than AB or EC (Table 1). Therefore, we expected to observe RFDMIA PI ΔRFU_Δ30min_ values where its first significantly increased PI ΔRFU_Δ30min_ value would occur at or close to its MIC, or at least discriminate susceptible from tolerant QAC concentrations by comparing PI ΔRFU_Δ30min_ values increases as we observed for EC and AB. However, when we compared PA PI ΔRFU_Δ30min_ values we detected high PI ΔRFU_Δ30min_ values at our lowest measured QAC concentration (9.4 µg/mL CET or BZK), which we know is well below PA's respective MIC of 75 µg/mL BZK or 150 µg/mL CET (Fig. 5A,B). This finding suggests that the PI dye used herein may not be able to accurately discriminate susceptible from tolerant QAC concentrations in PA based on monitoring significant increases in PI ΔRFU_Δ30min_ values. Since *Pseudomonas* spp. are known to have intrinsic fluorescent properties, we repeated this analysis to determine if another impermeant dye (SYTOX Blue) could improve *P. aeruginosa* RFDMIA results. SYTOX Blue has an EX/EM (444 nm/480 nm) value outside the nm range of PI (EX 544 nm/ EM 620 nm) potentially making it a feasible substitute for *P. aeruginosa* RFDMIA. We found that SYTOX Blue dye was also significantly under-predicted the QAC susceptibility of PA based on MIC or by using the lowest measured QAC concentration due its high background PI ΔRFU_Δ30min_ values even at sub-MIC BZK or CET concentrations (Fig. S5A,B). As a control, we also repeated RFDMIA with EC, where the lowest significantly increased SYTOX Blue ΔRFU_Δ30min_ value occurred at 18.8 µg/mL BZK and 37.5 µg/mL CET as observed for PI RFDMIA (Fig. S5). Therefore, SYTOX Blue or PI are both capable of being used for RFDMIA to discriminate QAC susceptibilities of species that do not have high intrinsic fluorescence such as *E. coli*, but not for intrinsically fluorescent *P. aeruginosa* species.

### RFDMIA can discriminate the susceptibility of unadapted and BZK-adapted *S. sonnei* and *K. pneumoniae* isolates

Lastly, to verify that other Enterobacterales species could be examined by RFDMIA, we applied RFDMIA to examine the QAC susceptibility of BZK-adapted *S. sonnei* (SSBZKT) and *K. pneumoniae* (KPBZKT) species. Both species were BZK adapted using the same experimental subculture gradual exposure method we used for *E. coli* ECCETT and ECBZKT. We generated three isolates from the BZK gradual exposure experiments that were included in this analysis. As we observed for QAC-adapted *E. coli* RFDMIAs, both SSBZKT and KPBZKT PI ΔRFU_Δ30min_ values could be used to discriminate BZK susceptible from tolerant isolates when compared to their respective unadapted *S. sonnei* (SS) or *K. pneumoniae* (KP) controls at or below their BZK MIC concentration tested (Fig. 5C,D). For KP RFDMIA, both its MIC (at 18.8 µg/mL BZK) and its 30MBC (at 37.5 µg/mL) were underpredicted when monitoring the first significant increase in PI ΔRFU_Δ30min_ value and maximum PI ΔRFU_Δ30min_ values respectively (Fig. 5C). The first significant increase in PI ΔRFU_Δ30min_ values for KPBZKT coincided with its MIC value (75.0 µg/mL BZK; Fig. 5C). As we observed for EC to ECBZKT RFDMIA comparisons, a noticeable difference in PI ΔRFU_Δ30min_ values at sub-MIC and MIC BZK concentrations was detected between KP and KPBZKT, indicating that BZK susceptible KP and BZK tolerant KPBZKT could be differentiated by RFDMIA at multiple BZK concentrations (Fig. 5C).

For SS RFDMIA, the first significant increase in PI ΔRFU_Δ30min_ value occurred at 9.4 µg/mL BZK which coincided with its MIC value but the maximum PI ΔRFU_Δ30min_ value did not coincide with its 30MBC value of SS at 18.8 µg/mL BZK; the SS RFDMIA maxima occurred at 150 µg/mL BZK (Fig. 5D; Table 1). Similar to SS, RFDMIA the first significant increase in PI ΔRFU_Δ30min_ value of SSBZKT coincided with its respective MIC value (at 37.5 µg/mL BZK; Fig. 5D). In contrast to SS RFDMIA, the BZK 30MBC value of SSBZKT also coincided with the maximum PI ΔRFU_Δ30min_ value (75 µg/mL BZK; Fig. 5D, Table 1). When comparing SSBZKT to SS PI ΔRFU_Δ30min_ values from 18.8 µg/mL BZK to 300 µg/mL BZK, we were able to differentiate the BZK susceptible from the tolerant isolate (Fig. 5D). Based on all of the RFDMIA results collected for various species and isolate, we developed a rapid fluorescent assay technique useful for discriminating the QAC antiseptic susceptibilities around MIC concentrations to accurately predict the MIC value of the species but, unable to consistently.

## Discussion

While investigating the robustness of RFDMIA to discriminate QAC susceptibility, we determined that different growth conditions influence RFDMIA detection accuracy. By comparing different growth physiologies we identified that stationary phase cell preparations gave RFDMIA results with lower error as compared to mid-log phase and colony cell preparations. There are a few explanations for why stationary phase cell preparations may be optimal for this assay. First, differences in cell envelope composition, capsule thickness^36,37^, lipid composition^38,39^, and membrane protein alterations^37^ have all been noted to differ among mid-log, colony, and stationary phase cells^40^. Since QACs primarily act by disrupting and micellarizing cell membrane lipids, differences in bacterial membrane lipid compositions may be an important factor influencing PI dye permeation in RFDMIAs. Previous studies have shown that the phospholipid composition of the bacteria grown to mid-log or grown on a solid media differs when comparing the quantity and ratios of phosphatidylethanolamine, phosphatidylglycerol, and cardiolipin^38,39^. This argument is supported by a recent in-depth characterization of ECCETT and ECBZKT isolates used herein reported in a study currently under review (bioRXIV# 201814), which identified alterations in lipid A, phospholipid biosynthesis, and transport systems. Bacterial cells grown as colonies also secrete additional extrapolymeric substances that prevent their desiccation when growing on solid surface to air interfaces, and also aid bacteria in adhering to the solid surface^41,42^. The differences in lipids and secreted substances from colonies, and/or perhaps the clumping of colony cells when resuspended into PBS buffer for RFDMIA, likely obscure the accurate prediction of QAC susceptibility based on RFDMIA PI ΔRFU_Δ30min_ values. It is important to note that only a few discrepancies were noted in QAC susceptibility predictions determined by RFDMIA for mid-log and stationary phase *E. coli* isolate preparations, primarily in PI ΔRFU_Δ30min_ value error. Hence, stationary phase cell preparations are ideal for this rapid fluorescent screening technique to detect QAC susceptibility.

In our study, the lowest QAC concentrations we measured corresponded to sub-inhibitory MIC concentration of each isolates. It is likely that RFDMIA is sensitive enough to examine lower concentrations of QAC and in future studies should explore these limits. We have shown that RFDMIA does have a maximum detection threshold, which is most likely associated to the CMC and/or the counter ion of the QAC employed. The high QAC concentrations used in our study correspond to previously reported CET and BZK CMC values of 750 and 100 μg/ml respectively^32–35^. The co-occurrence of PI RFU_30min_ EM signal loss as QAC CMC values were reached by RFDMIA (Figs. 1, 5, S1E,F, S2) suggests that QAC micellization prevents accurate detection at high QAC concentrations by RFDMIA. Additionally, high QAC concentrations also increase the concentration of anionic counter ions (Cl^−^ and Br^−^) present in solution which may also increase signal quenching^43^, however we did not see significant increases in quenching at high QAC concentrations in the presence of DNA and PI (Fig. S2E,F). Since PI is a structural analog of ethidium bromide, increased counter anion concentrations may impact EM signal intensity, may contribute to dye RFU EM signal loss, and increase signal saturation in live versus heat-treated samples tested in our analysis (Figs. S1, S2E,F). Overall, the range of QAC concentrations we measured in this analysis were sufficient to discriminate between nearly all of the QAC-susceptible and tolerant isolates we examined, indicating this assay is worth pursuing further as an antiseptic/disinfectant specific assay in future applications.

In our study, we relied on the use of QAC-adapted isolates as there are currently no well-established QAC tolerant reference strains available for testing, and this resulted in the unexpected finding of QAC dependence using RFDMIA and SEM analyses. QAC-adapted ECBZKT and ECCETT resuspended in PBS without added QAC resulted in cells with flattened or deflated cell morphologies based on SEM visualization. Using the same SEM fixation conditions, the parental EC maintained the anticipated turgid bacilliform morphology. Once exposed to QACs at sub-MIC concentrations, the morphology of both QAC-adapted *E. coli* isolates appeared to re-inflate similar to the unadapted non-QAC exposed EC (Tables S1, S4). This was especially evident for the chain forming ECCETT which had greater proportions of inflated cells as CET concentrations fell within sub-MIC values (Fig. 4; Table S4). For ECBZKT isolates when BZK was introduced, the proportion of inflated and intermediate cells did slightly increase, though to a lesser degree than ECCETT (Tables S1, S4). Interestingly, instead of the development of chains, ECBZKT showed an elongated phenotype, being on average, across all concentrations, 0.402 µm longer but − 0.172 µm thinner than EC. These morphological alterations have not been reported for QAC-adapted *E. coli* isolates in previous studies to date, making this a novel finding. Based on the few studies that do examine electron microscopy images of bacteria exposed to QACs, most indicate that the cell membrane of the imaged bacterium (*Pseudomonas fluorescens*^44^, *P. aeruginosa*^44–46^, *Staphylococcus aureus*^47^, and *E. coli*^47^) is stripped off in the presence of QACs, which we did not see in our EC SEM images (Figs. 3, 4), however, these studies only provided representative images and did not include blinded analyses of multiple cell counts. Together, this study shows additional applications for the RFDMIA to not only predict QAC tolerance of bacteria but also to probe deeper into cell membrane permeability using impermeant fluorescent dyes.

Our RFDMIA results often underpredicted MIC values for QAC-adapted Enterobacterial isolates. This may be due to the differences in cell morphology caused by QAC adaptation versus unadapted isolates we observed in our study. Altered cell morphology due to prolonged QAC adaptation likely increases *E. coli* permeability to the impermeant dye due to altered membrane compositions, noted in previous studies^31,40^ and from our own isolate characterization (bioRXIV# 201814). The underprediction of MIC values by RFDMIA is most likely due to significant differences in methodologies and cell physiologies used in AST versus RFDMIA. AST measures cell susceptibility as an MIC value at the lowest concentration of drug that prevents cell growth in media over an 18–24 h period, whereas RFDMIA takes stationary phase cells in PBS and exposes them to drug over a 30-min timeframe. Although both methods involve stationary phase cells, neither method is directly comparable per se, making the 30MBC values a better estimate of cell viability for RFDMIA. Hence, additional RFDMIA experiments involving QAC-adapted *E. coli* at lower QAC concentrations (at and far below their MIC) may be necessary to determine if longer exposure times more accurately predict MIC values from RFU values. RFDMIA may have also been influenced by QAC-adapted *E. coli* exhibiting a QAC dependence. When low or no QAC concentrations were not to QAC-adapted *E. coli* suspensions, it may detrimentally affect cell membrane integrity, altering PI dye uptake, and result in underprediction of MIC values as observed by SEM imaging. Lastly, QAC-adapted *E. coli* tested in this study were known to have numerous genetic alterations that impacted multiple QAC mechanisms of tolerance and membrane composition (bioRXIV# 201814). Many altered genes identified in these isolates impacted three of the known mechanisms of antiseptic tolerance, efflux alterations, lipid A alterations and porin downregulation^4,11,29^. It is unclear if altered mechanisms besides lipid alterations, impact impermeant fluorescent dye penetration, and this will be explored further in future studies.

Using RFDMIA, we were able to discriminate QAC susceptibilities of AB towards CET and BZK, SS to BZK, as well as KP to BZK. However, RFDMIA was unable to accurately predict the QAC susceptibility of PA for either BZK (Fig. 5A; Fig. S5A) or CET (Fig. 5B; Fig. S5B) independent of the fluorescent dye employed (PI or SYTOX Blue). This result is likely due to the fact that *P. aeruginosa* and other fluorescent Pseudomonads are capable of producing a variety of fluorescently active pigments. One compound that is enriched in *P. aeruginosa* species is a chromophore molecule known as aeruginosin A^48,49^. Aeruginosin A has similar spectral overlap as PI dye with maximum EM ranges occurring between 600 and 650nm^49^. The overlap in EM likely explains why *P. aeruginosa* RFDMIA PI ΔRFU_Δ30min_ values we detected were much higher than any other isolate tested by RFDMIA. Our use of Sytox Blue as an alternative impermeant fluorescent dye for RFDMIA, was also incapable of discriminating QAC susceptibility likely due to the high fluorescent background emission contributions from other pigments produced by *P. aeruginosa*, as demonstrated in previous studies^50^. Further investigation into alternative impermeant dyes as RFDMIA detection methods may be a detection solution for our assay. For now, the fluorescent properties of *P. aeruginosa* and other intrinsically fluorescent bacterial species will limit the applicability of RFDMIA.

In conclusion, the results presented herein illustrate the applications of using RFDMIA as a rapid screening tool for measuring QAC susceptibilities in a short amount of time. We have shown that RFDMIA can detect bacterial QAC susceptibility in Enterobacterales sensitively and within value ranges used to estimate MIC values by AST methods (Fig. 1). RFDMIA is can do this rapidly using stationary phase cells (Fig. 4), and for most species we tested (Fig. 5). The sensitivity of RFDMIA at sub-MIC concentrations shows that we were able to differentiate between QAC-susceptible isolates and their QAC-tolerant counterparts. We were also able to use a relatively low concentration of CET or BZK (10–40 µg/mL) to differentiate between QAC-tolerant and susceptible *E. coli* isolates. The only limitation for this assay is its ability to detect susceptibility of intrinsically fluorescent bacteria like Pseudomonads, especially those with EX/EM spectral overlap that are similar to the assay’s dye. The assay is also limited to QAC concentrations below its respective CMC due to the inherent micelle forming properties of detergent-like QACs. Lastly, our SEM images revealed a type of QAC dependence by CET adapted *E. coli* that had not been previously seen and highlight cell morphology perturbations and the consequences of QAC adaptation resulting in increased QAC tolerance.

## Materials and methods

### Chemicals used in the study

Benzalkonium chloride (BZK, 12,060) was purchased from Millipore Sigma (USA). Alkyltrimethylammonium bromide (Cetrimide; CET, M7635) was purchased from Fisher Scientific (USA). PI (EX/EM: 544 nm/620 nm; P3566) and SYTOX Blue (EX/EM: 444 nm/480 nm; S34857) were purchased from Invitrogen (USA).

### Bacterial isolates and culture conditions

All species and isolates tested in this study are listed in Table 1. Three *E. coli* K-12 BW25113 isolates were selected to validate the RFDMIA, a parental BW25113 (EC) strain, and two laboratory adapted isolates derived from EC that were repeatedly sub-cultured with either BZK (ECBZKT) or CET (ECCETT) to obtain isolates with fourfold increased QAC MIC values. Analysis of the QAC-adapted *E. coli* used in our study was described in a recently submitted study under review (bioRXIV# 201814). The same method used to gradually adapt *E. coli* was used to adapt *S. sonnei* (SSBZKT) and *K. pneumoniae* (KPBZKT) to BZK as listed in Table 1. All isolates were grown in Luria Bertani (LB) broth at 37 °C in a shaking incubator (170 rpm). QAC-adapted isolates (ECCETT, ECBZKT, SSBZKT, KPBZKT) were grown with added CET or BZK (50 µg/mL CET; 40 µg/mL BZK, 10 µg/mL BZK, 28 µg/mL BZK) respectively, to maintain QAC-tolerant phenotype selection.

### RFDMIA fluorescent spectrophotometry

#### Optimal fluorescent dye and cell concentration determination

To determine the appropriate concentration of PI RFDMIAs, an initial fluorescent emission checkerboard assay was performed to optimize dye and cell concentrations. Checkerboard assays were performed in polystyrene optical bottom black-walled fluorescent 96-well microplates (265,301, Thermo Scientific, USA) with increasing PI concentrations (0–10 µg/mL) incubated with increasing concentrations of genomic DNA extracted from *E. coli* cultures grown to 0, 0.05, 0.1, 0.2, 0.4 OD_600nm_ units, or live EC cell suspensions at OD_600nm_ values 0, 0.05, 0.1, 0.2, 0.4 units, or heat-treated EC cells at OD_600nm_ values of 0, 0.05, 0.1, 0.2, 0.4 units (Fig. S2A–D). All cells used for fluorescent analyses were resuspended in 0.2 µm filtered (CA28145-501, VWR, Canada) phosphate buffered saline (PBS). Fluorescence excitation and emission (EX 544 nm ± 20 nm; EM 620 nm ± 10 nm) was performed in a Polarstar Optima fluorescent microplate spectrophotometer (BMG Labtech, Germany). Based on results of the checkerboard assay, we selected live cell suspensions of OD_600nm_ = 0.2 units and 2.0 µg/mL PI dye, as this concentration was optimal to detect a reasonable amount of dye emission from the lowest amount of cells added (Fig. S2A; black arrow). For assays involving SYTOX Blue, we followed the recommended manufacturer concentration of 1 µM final concentration.

#### RFDMIA cell culture preparations

Prior to RFDMIA experiments, cell culture preparation was performed to standardize cells using the following protocol. Cryo-preserved stocks (in 16% glycerol final concentration) of each isolate were grown in 10 mL LB as three biological replicates overnight (18 h). OD_600nm_ values of each culture were measured by spectrophotometer (DU530, Beckman and Coulter, USA) and standardized to a final OD_600nm_ value of 2.0 units. Standardized stationary phase cultures were divided into samples used for live cell measurements and heat-treated control sample preparations (Fig. S1). Divided cell samples were centrifuged for 2 min at 14,000 rpm, washed twice with filtered PBS, resuspended in filtered PBS and stored on ice until aliquoted for the RFDMIA. Heat-treated samples were placed in a heating block at 121 °C for 30 min. Suspensions of live and heat-treated samples were diluted with filtered PBS to achieve a final bacterial suspension with OD_600nm_ = 0.2 units. For mid-log samples, overnight cultures were diluted 1:50 into 10 mL of fresh LB with selection if needed and left to grow to mid-log in a shaking incubator. Once mid-log (OD_600nm_ = 0.5 units) was achieved, the cells were treated in the exact same manner as the stationary phase preparation. For colony samples, 50 µl of overnight culture was spread plated onto agar containing selection if necessary and left to grow overnight. After, the bacteria were scraped into filtered PBS and standardized to OD_600nm_ of 2.0 units then were treated the same way as stationary and mid-log cells.

#### RFDMIA protocol

Optical bottom black fluorescent 96-well microplates (265,301, Thermo Scientific, USA) were used for RFDMIA. Each plate well contained PI at a final concentration of 2 µg/mL and each column contained increasing concentrations of BZK or CET (0–600 µg/mL; twofold dilution) unless otherwise indicated. 100 µL of either filtered PBS (blank) or standardized bacterial resuspension (heat-treated or sample) was added to each microplate well. Microplates were measured in a fluorescent microplate-reader (Polarstar Optima, BMG labtech, Germany) where each well was monitored every five minutes for 30-min. After the assay was complete, a 30-min spot-plate viability was performed using the 30-min MBC AST procedure detailed in Sect. 2.4.2.

#### RFDMIA EM RFU calculations

This analysis involved three step-wise calculations (Eqs. 1–3) to measure the change in RFUs after 30 min (_Δ_RFU_30min_) at a given X µg/mL QAC concentration.

1 $$\begin{aligned} {\text{T}}_{{0{\text{min}}}} & = {\text{ RFU}}_{{{\text{Sample}}}} {-}{\text{ RFU}}_{{{\text{Blank}}}} = {\text{ RFU}}_{{0{\text{min }}[{\text{X}}\mu {\text{g}}/{\text{mL QAC}}]}} \\ {\text{T}}_{{{3}0{\text{min}}}} & = {\text{ RFU}}_{{{\text{Sample}}}} {-}{\text{ RFU}}_{{{\text{Blank}}}} = {\text{ RFU}}_{{{3}0{\text{min }}[{\text{X}}\mu {\text{g}}/{\text{mL QAC}}]}} \\ \end{aligned}$$

Equation 1 calculates the sample RFU values at a given EM wavelength at the start (T_0min_) and the end (T_30min_) of the assay incubation (RFU_0mins_ and RFU_30mins_), by subtracted the blank RFU values from wells containing only dye at each X µg/mL QAC (RFU_Blank_) from the live cell suspension samples at the same X µg/mL QAC concentration (RFU_Sample_); where “X” designates a defined concentration of QAC (µg/mL).

2 $${\text{RFU}}_{{{3}0{\text{min}}}} - {\text{ RFU}}_{{0{\text{min}}}} = {\text{ RFU}}_{{\Delta {3}0{\text{min }}\left[ {{\text{X}}\mu {\text{g}}/{\text{mL QAC}}} \right]}}$$

Equation 2 measures the difference in RFU values for an isolate at the same QAC concentration over 30-min (RFU_Δ30min [Xµg/mL QAC]_), by subtracting the RFU_30min [Xµg/mL QAC]_ from the RFU_0min [Xµg/mL QAC]_.

3 $${\text{RFU}}_{{\Delta {3}0\;{\text{min }}[{\text{X}}\;\upmu {\text{g}}/{\text{mL QAC}}]}} - {\text{ RFU}}_{{\Delta {3}0{\text{min }}[0\;\upmu {\text{g}}/{\text{mL QAC}}]}} = \, \Delta {\text{RFU}}_{{\Delta {3}0{\text{min}}}}$$

Lastly, Eq. 3 is used to control for any fluorescent dye uptake by the isolate without QAC exposure (0 µg/mL QAC (RFU_Δ30min [0 µg/mL QAC]_). For this, we subtracted RFU_Δ30min [0 µg/mL QAC]_ from RFU_Δ30min [Xµg/mL QAC]_.

### Antimicrobial susceptibility testing (AST)

#### Broth microdilution AST for MIC calculations

Broth microdilution AST were conducted as described by Balouiri et al*.*^51^ to determine MIC values for all isolates against BZK and/or CET. Briefly, cryopreserved stocks of each isolate were grown overnight (18 h) and the next day, the OD_600nm_ of the cultures were measured using a spectrophotometer (DU530, Beckman and Coulter, USA) and adjusted to 1.0 units with LB broth. Standardized cultures were diluted to 0.2 × 10^–4^ units into 96-well microplates (167,008, Thermo Scientific, USA) containing LB and increasing concentrations of BZK or CET. After inoculation, the 96-well microplates were incubated overnight. After incubation, OD_600nm_ were measured with an Ultraviolet/Visible wavelength plate spectrophotometer (Multiskan spectrum, Fisher Scientific, USA). The MIC value was defined by the lowest concentration of antimicrobial where there was no discernable growth (OD_600nm_) from the blank well. MIC values were based on 3 biological replicates and at least 3 technical replicates. Broad range MIC values as well as narrow range/step-up MIC values were determined separately to coincide with their appropriate RFDMIA (Figs. 1, 2, 5, S5).

#### 30-min MBC (30MBC) determination

A 30MBC for each bacterium tested using the RFDMIA was measured using an LB agar spot plating method to determine each isolate’s viability after QAC exposure. This method involved LB agar spotting approximately 1–2 µL of each bacterial suspension per well of a RFDMIA microplate after 30-min incubation using a sterilized 48-pin replicator (05-450-10, Boekel Scientific, USA). QAC-adapted isolates (ECCETT, ECBZKT, SSBZKT, KPBZKT) were with spotted onto LB agar with QAC selection when necessary (50 µg/mL CET; 40 µg/mL BZK, 10 µg/mL BZK, 28 µg/mL BZK). Spotting was performed at a minimum of technical triplicate per biological replicate and the spotted plates were incubated at 37 °C overnight. We defined the 30MBC value as the mean of the lowest QAC concentration at which no bacterial growth occurred on the agar spot plate based on at least three spotted replicates of the 3 technical and 3 biological replicates.

### Scanning electron microscopy (SEM)

We utilized scanning electron microscopy (SEM) JCM-5700 instrument (JEOL USA, USA) to visualize *E. coli* cell morphology after strict 18 h growth to verify alterations to *E. coli* isolates at various QAC concentrations used for RFDMIA. Bacterial samples were prepared and resuspended in PBS using the same protocol as described in 2.3.1 for the RFDMIA. Cell QAC exposures were strictly limited to 30-min and then immediately fixed for SEM analysis. SEM imaging of bacterial PBS suspensions followed the gold sputtering protocol described by Golding et al*.*^52^, with a modification that we diluted bacteria 1:1000. Five separate images at 5000× magnification were collected at each QAC concentration for each respective isolate measured. To select representative images shown in Figs. 3 and 4, each of the five image sets per QAC concentration was blinded according to isolate and QAC concentration, and images were assessed for the number of deflated, intermediate, and inflated cells in addition to measure cell width and lengths from five different areas of each image. Brightness and contrast standardization of SEM images at each concentration of QAC was also performed and all image analysis was completed using ImageJ V1.52a^53^. With this software, 20 lengths and 20 widths were measured (in μm) per image from each of the five images for each isolate (n = 100/isolate) to assess differences in morphology and determine cell deflation. Further, to assure an unbiased interpretation of what constituted “inflation”, “intermediate” and “deflated” cell morphologies, images were blinded and subsequently analyzed by two separate researchers/ assessors who were given identical instructions and a template image of what would qualify as inflated, intermediate, and deflated.

### Statistical anaslysis

All statistical analyses were performed using GraphPad Prism V6 (GraphPad Software, USA) or Excel365 (Microsoft, USA). For RFDMIA analysis, we performed two two-tailed Student’s *t* tests to analyze all ΔRFU_Δ30min_ EM data. The first was comparing a given concentration of QAC to the lowest QAC concentration and considered changes with *P* values of < 0.05 to be considered significant. The second was comparing the ΔRFU_Δ30min_ EM values between isolates within a given RFDMIA to distinguish between the varying BZK or CET tolerance. Unless otherwise noted, RFDMIA values shown in all figures represent the mean ΔRFU_Δ30min_ value of the dye at its respective EM value and was determined from the mean of 3 technical replicates per biological replicate (n = 3) of each isolate. For inflation/deflation SEM image analysis, we performed a non-parametric Mann Whitney U test to determine significantly different values between no drug added to drug exposed isolate samples (indicated by *) or between unadapted to adapted isolate values (indicated by †); for both comparisons *P* < 0.05 was considered significant.

## Supplementary information

Supplementary Information.

## References

[1] Gerba CP (2015). Quaternary ammonium biocides: Efficacy in application. Appl. Environ. Microbiol..

[2] Rutala, W. A. & Weber, D. J. 301 - Disinfection, Sterilization, and Control of Hospital Waste. In *Mandell, Douglas, and Bennett’s Principles and Practice of Infectious Diseases* 3294–3309.e4 (Elsevier Inc, 2015). 10.1016/B978-1-4557-4801-3.00301-5.

[3] Zou L (2014). Presence of disinfectant resistance genes in *Escherichia coli* isolated from retail meats in the USA. J. Antimicrob. Chemother..

[4] Tezel U, Pavlostathis SG (2015). Quaternary ammonium disinfectants: Microbial adaptation, degradation and ecology. Curr. Opin. Biotechnol..

[5] Negin C, Ali S, Xie Q (2017). Most common surfactants employed in chemical enhanced oil recovery. Petroleum.

[6] Buffet-Bataillon S, Le Jeune A, Le Gall-David S, Bonnaure-Mallet M, Jolivet-Gougeon A (2012). Molecular mechanisms of higher MICs of antibiotics and quaternary ammonium compounds for *Escherichia coli* isolated from bacteraemia. J. Antimicrob. Chemother..

[7] Wales A, Davies R (2015). Co-selection of resistance to antibiotics, biocides and heavy metals, and its relevance to foodborne pathogens. Antibiotics.

[8] Gilbert P, McBain AJ (2003). Potential impact of increased use of biocides in consumer products on prevalence of antibiotic resistance. Clin. Microbiol. Rev..

[9] Bloomfield SF (2002). Significance of biocide usage and antimicrobial resistance in domiciliary environments. J. Appl. Microbiol. Sympos. Suppl..

[10] Hegstad K (2010). Does the wide use of quaternary ammonium compounds enhance the selection and spread of antimicrobial resistance and thus threaten our health?. Microb. Drug Resist..

[11] Gilbert P, Moore LE (2005). Cationic antiseptics: Diversity of action under a common epithet. J. Appl. Microbiol..

[12] Jennings MC, Minbiole KC, Wuest WM (2015). Quaternary Ammonium Compounds: An Antimicrobial Mainstay and Platform for Innovation to Address Bacterial Resistance. ACS Infect. Dis..

[13] Lee JY, Wang B-J (1995). Contact dermatitis caused by cetrimide in antiseptics. Contact Dermat..

[14] Buffet-Bataillon S, Tattevin P, Bonnaure-Mallet M, Jolivet-Gougeon A (2012). Emergence of resistance to antibacterial agents: The role of quaternary ammonium compounds—a critical review. Int. J. Antimicrob. Agents.

[15] Zhang X, Ma J, Chen M, Wu Z, Wang Z (2018). Microbial responses to transient shock loads of quaternary ammonium compounds with different length of alkyl chain in a membrane bioreactor. AMB Express.

[16] Epand RM, Epand RF (2009). Lipid domains in bacterial membranes and the action of antimicrobial agents. Biochim. Biophys. Acta Biomembr..

[17] Delcour AH (2009). Outer membrane permeability and antibiotic resistance. Biochim Biophys Acta..

[18] Davin-Regli A, Pagès JM (2012). Cross-resistance between biocides and antimicrobials: An emerging question. Rev. Sci. Tech..

[19] World Health Organization. *Global Priority List of Antibiotic-Resistant Bacteria to Guide Research, Discovery, and Development of New Antibiotics*. https://www.who.int/medicines/publications/WHO-PPL-Short_Summary_25Feb-ET_NM_WHO.pdf (2017). 10.1016/S1473-3099(09)70222-1.

[20] Romero, J. L., Grande Burgos, M. J., Pérez-Pulido, R., Gálvez, A. & Lucas, R. Resistance to Antibiotics, Biocides, Preservatives and Metals in Bacteria Isolated from Seafoods: Co-Selection of Strains Resistant or Tolerant to Different Classes of Compounds. *Front. Microbiol.* **8**, 1–16 (2017). 10.3389/fmicb.2017.01650 PMC5583239 28912764

[21] Aulawi K, Junko N, Okada S (2018). Relationship between resistance to antibiotics and insusceptibility to biocides of *Staphylococcus aureus* and *Pseudomonas aeruginosa* isolated in Indonesian hospitals. Int. J. Res. Med. Sci..

[22] Braoudaki M, Hilton AC (2004). Adaptive resistance to biocides in *Salmonella enterica* and *Escherichia coli* O157 and cross-resistance to antimicrobial agents. J. Clin. Microbiol..

[23] Tezel U, Pavlostathis SG, Keen PL, Montforts MHMM (2011). Role of quaternary ammonium compounds on antimicrobial resistance in the environment. Antimicrobial Resistance in the Environment.

[24] Van Boeckel TP (2014). Global antibiotic consumption 2000 to 2010: An analysis of national pharmaceutical sales data. Lancet Infect. Dis..

[25] Sheldon AT (2005). Antiseptic ‘resistance’: Real or perceived threat?. Clin. Infect. Dis..

[26] O’Driscoll NH, Cushnie TPT, Matthews KH, Lamb AJ (2018). Colistin causes profound morphological alteration but minimal cytoplasmic membrane perforation in populations of *Escherichia coli* and *Pseudomonas aeruginosa*. Arch. Microbiol..

[27] Cerf O, Carpentier B, Sanders P (2010). Tests for determining in-use concentrations of antibiotics and disinfectants are based on entirely different concepts: “Resistance” has different meanings. Int. J. Food Microbiol..

[28] Mcdonnell G, Russell AD (1999). Antiseptics and disinfectants: Activity, action, and resistance. Clin. Microbiol. Rev..

[29] Langsrud S, Sidhu MS, Heir E, Holck AL (2003). Bacterial disinfectant resistance - A challenge for the food industry. Int. Biodeterior. Biodegrad..

[30] Russell AD (2004). Bacterial adaptation and resistance to antiseptics, disinfectants and preservatives is not a new phenomenon. J. Hosp. Infect..

[31] Bore E (2007). Adapted tolerance to benzalkonium chloride in *Escherichia coli* K-12 studied by transcriptome and proteome analyses. Microbiology.

[32] Shanthi PMSL (2010). Synthesis and characterization of porous shell-like nano hydroxyapatite using cetrimide as template. J. Colloid Interface Sci..

[33] Duquemin SJ, Nixon JR (1985). The effect of sodium lauryl sulphate, cetrimide and polysorbate 20 surfactants on complex coacervate volume and droplet size. J. Pharm. Pharmacol..

[34] Georgiev GA (2011). Surface chemistry study of the interactions of benzalkonium chloride with films of meibum, corneal cells lipids, and whole tears. Invest. Ophthalmol. Vis. Sci..

[35] Kato Y, Yagi H, Kaji Y, Oshika T, Goto Y (2013). Benzalkonium chloride accelerates the formation of the amyloid fibrils of corneal dystrophy-associated peptides. J. Biol. Chem..

[36] Cunnion KM, Lee JC, Frank MM (2001). Capsule production and growth phase influence binding of complement to *Staphylococcus aureus*. Infect. Immun..

[37] Navarro Llorens JM, Tormo A, Martínez-García E (2010). Stationary phase in Gram-negative bacteria. FEMS Microbiol. Rev..

[38] Rowlett VW (2017). The impact of membrane phospholipid alterations in *Escherichia coli* on cellular function. J. Bacteriol..

[39] Salanitro JP, Wegener WS (1971). Growth of *Escherichia coli* on short-chain fatty acids: Growth characteristics of mutants. J. Bacteriol..

[40] Whitfield C, Roberts IS (1999). Structure, assembly and regulation of expression of capsules in *Escherichia coli*. Mol. Microbiol..

[41] Gilbert P, Evans DJ, Evans E, Duguid IG, Brown MRW (1991). Surface characteristics and adhesion of *Escherichia coli* and *Staphylococcus epidermidis*. J. Appl. Bacteriol..

[42] Bales PM, Renke EM, May SL, Shen Y, Nelson DC (2013). Purification and characterization of biofilm-associated EPS exopolysaccharides from ESKAPE organisms and other pathogens. PLoS ONE.

[43] Geddes CD (2001). Optical halide sensing using fluorescence quenching: Theory, simulations and applications—a review. Meas. Sci. Technol..

[44] Langsrud S, Sundheim G, Borgmann-Strahsen R (2003). Intrinsic and acquired resistance to quaternary ammonium compounds in food-related *Pseudomonas* spp. J. Appl. Microbiol..

[45] Rutala WA, Stiegel MA, Sarubbi FA, Weber DJ (1997). Susceptibility of antibiotic-susceptible and antibiotic-resistant hospital bacteria to disinfectants. Infect. Control Hosp. Epidemiol..

[46] Washam CJ, Sandine WE, Elliker PR (1976). A strain of *Pseudomonas aeruginosa* resistant to a quaternary ammonium compound. J. Milk Food Technol..

[47] Ma J (2019). Synthesis, physicochemical and antimicrobial properties of cardanol-derived quaternary ammonium compounds (QACs) with heterocyclic polar head. J. Mol. Liq..

[48] Meyer JM (2000). Pyoverdines: Pigments, siderophores and potential taxonomic markers of fluorescent pseudomonas species. Arch. Microbiol..

[49] Abu EA (2013). Cyclic voltammetric, fluorescence and biological analysis of purified aeruginosin A, a secreted red pigment of *Pseudomonas aeruginosa* PAO1. Microbiology.

[50] Wasserman AE (1965). Absorption and fluorescence of water-soluble pigments produced by four species of *Pseudomonas*. Appl. Microbiol..

[51] Balouiri M, Sadiki M, Ibnsouda SK (2016). Methods for in vitro evaluating antimicrobial activity: A review. J. Pharm. Anal..

[52] Golding CG, Lamboo LL, Beniac DR, Booth TF (2016). The scanning electron microscope in microbiology and diagnosis of infectious disease. Sci. Rep..

[53] Abràmoff MD, Magalhães PJ, Ram SJ (2007). Image Processing with ImageJ. Biophotonics Int..

