# Supplementary material for: Antiseptic quaternary ammonium compound tolerance by gram-negative bacteria can be rapidly detected using an impermeant fluorescent dye-based assay
Source: Sci Rep. 2020 Nov 25;10:20543. doi: 10.1038/s41598-020-77446-8 (PMC7689532; doi:10.1038/s41598-020-77446-8)
Supplement: Supplementary file 1 — Supplementary Information. [file 41598_2020_77446_MOESM1_ESM.pdf]

SUPPLEMENTARY FILES

For the research manuscript entitled:

Antiseptic quaternary ammonium compound tolerance by Gram-negative bacteria can be rapidly detected using an impermeant fluorescent dye-based assay

By authors: Branden S. J. Gregorchuk[1], Shelby L. Reimer[1], Daniel R. Beniac[2], Shannon L. Hiebert[2], Timothy F. Booth[1,2], Michelle Wuzinski[1], Brielle E. Funk[1], Kieran A. Milner[1], Nicola H. Cartwright[1], Ali N. Doucet[1], Michael R. Mulvey[2], Mazdak Khajehpour[3], George G. Zhanel[1], Denice C. Bay[1§]

1. Department of Medical Microbiology and Infectious Diseases, University of Manitoba, Winnipeg, Manitoba, Canada
2. National Microbiology Laboratory, Public Health Agency of Canada, Winnipeg, Manitoba, Canada
3. Department of Chemistry, University of Manitoba, Winnipeg, Manitoba, Canada

[§]Corresponding author:

Assistant Professor
Rm 514C Basic Medical Sciences Bldg.
Department of Medical Microbiology and Infectious Diseases
University of Manitoba
745 Bannatyne Avenue
Winnipeg, MB, Canada R3E 0J9
Tel: (204) 977-5679
Fax: (204) 789-3926
Email: Denice.Bay@umanitoba.ca

Table S1. Mean length and width values of EC and ECBZKT after 30-minute exposure to different BZK concentrations [BZK] based on five SEM images at 5000 magnification (n=100).

[BZK] (µg/mL)	Isolate	Length (µm)	Standard deviation	Difference from EC[a]	Width (µm)	Standard deviation	Difference from EC[a]
0	EC	1.363	0.293		0.742	0.083	
	ECBZKT	1.781	0.543	**0.418**	0.651	0.070	**-0.091**
9.4	EC	2.205	0.551		0.958	0.095	
	ECBZKT	1.863	0.467	**-0.342**	0.672	0.072	**-0.286**
18.8	EC	1.313	0.280		0.921	0.095	
	ECBZKT	1.849	0.477	**0.536**	0.676	0.065	**-0.246**
75	EC	1.199	0.202		0.861	0.089	
	ECBZKT	1.874	0.528	**0.675**	0.723	0.114	**-0.138**
150	EC	1.061	0.198		0.818	0.072	
	ECBZKT	1.784	0.450	**0.723**	0.723	0.058	**-0.095**

All bolded values were significantly different from EC at *P* <0.01 by a two-tailed Students t-test.

[a]Each "Difference from EC" value was determined by subtracting mean length or width of EC from mean length or width values of ECCETT.

Table S2. Mean percentage of deflated, indeterminate, and inflated cell morphologies of EC and ECBZKT cells after 30-minute exposure to BZK at various concentrations.

[BZK] (µg/mL)		EC			ECBZKT		
		Deflated	Intermediate	Inflated	Deflated	Intermediate	Inflated
0.0	Avg	8.50%	17.00%	74.50%	27.00%†	26.50%†	46.50%†
	Stdev	0.71%	7.07%	6.36%	5.66%	3.54%	2.12%
9.4	Avg	82.00%*	18.00%	0.00%*	23.50%†	36.00%	40.50%†
	Stev	25.46%	25.46%	0.00%	12.02%	18.38%	6.36%
18.8	Avg	31.63%*	48.47%	19.90%*	18.50%	35.50%	46.00%†
	Sdev	31.75%	44.01%	12.27%	2.12%	4.95%	2.83%
75.0	Avg	18.50%	70.00%	11.50%	35.50%*	32.50%†	32.00%*†
	Stdev	19.09%	19.80%	0.71%	4.95%	9.19%	4.24%
150.0	Avg	19.77%	55.81%	24.42%*	19.50%*	43.50%*	37.00%†
	Stdev	11.51%	26.31%	14.80%	6.36%	3.54%	2.83%

Abbreviations: Avg; average, Stdev; standard deviation.

Stdev represents variation noted between the deflated, intermediate, and inflated cell proportions determined by of each researcher.

*: indicates significant ($P<0.05$; determined by Mann Whitney U test) change in abundance as compared to previous BZK concentration per isolate

†: indicates significant ($P<0.05$; determined by Mann Whitney U test) change in abundance between EC and ECBZKT at the specific [BZK]

All images are based on blinded cell counts from five SEM images at 5000X magnification (n=100) by two independent researchers.

Table S3. Mean length and width values of EC and ECCETT after 30-minute exposure to different CET concentrations [CET] based on five SEM images at 5000X magnification (n=100).

[CET] (µg/mL)	Isolate	Length (µm)	Standard deviation	Difference from EC[a]	Width (µm)	Standard deviation	Difference from EC[a]
0	EC	1.363	0.293	**0.294**	0.742	0.083	**0.120**
	ECCETT	1.657	0.429		0.862	0.120	
18.8	EC	1.205	0.247	**0.236**	0.808	0.126	**-0.051**
	ECCETT	1.441	0.306		0.757	0.104	
37.5	EC	1.242	0.259	**0.109**	0.811	0.094	**-0.182**
	ECCETT	1.351	0.286		0.629	0.070	
75	EC	1.153	0.266	**0.171**	0.797	0.075	**-0.132**
	ECCETT	1.324	0.316		0.665	0.066	
300	EC	1.342	0.388	0.063	0.912	0.106	**-0.161**
	ECCETT	1.405	0.305		0.751	0.130	

All bolded values were significantly different from EC at $P < 0.01$ by a two-tailed Students t-test.

[a]Each "Difference from EC" value was determined by subtracting mean length or width of EC from mean length or width values of ECCETT.

Table S4. Mean percentage of deflated, indeterminate, and inflated of EC and ECCETT cells after 30-minute exposure to CET at various concentrations.

[CET] (µg/mL)		EC			ECCETT		
		Deflated	Intermediate	Inflated	Deflated	Intermediate	Inflated
0.0	Avg	8.50%	22.00%	69.50%	68.28%†	20.00%	11.72%†
	Stdev	4.95%	14.14%	19.09%	17.99%	14.14%	3.85%
18.8	Avg	12.02%	31.14%	56.84%	20.00%*†	33.50%	46.50%*†
	Stdev	1.45%	2.08%	3.53%	0.00%	10.61%	10.61%
37.5	Avg	65.55%*	27.08%	7.37%*	3.00%*†	20.00%	77.00%*†
	Stdev	33.16%	22.74%	10.42%	4.24%	21.21%	25.46%
75.0	Avg	17.00%*	55.50%*	27.50%*	9.50%	31.50%†	59.00%†
	Stdev	1.41%	6.36%	7.78%	10.61%	6.36%	4.24%
300.0	Avg	67.50%*	27.50%*	5.00%*	36.50%*†	33.00%	30.50%*†
	Stdev	14.85%	13.44%	1.41%	6.36%	9.90%	3.54%

Abbreviations: Avg; average, Stdev; standard deviation.

Stdev represents variation noted between the deflated, intermediate, and inflated cell proportions determined by of each researcher.

*: indicates significant ($P<0.05$; determined by Mann Whitney test) change in abundance as compared to previous CET concentration per isolate.

†: indicates significant ($P<0.05$; determined by Mann Whitney test) change in abundance between EC and ECCETT at the specific [CET]

All images are based on blinded cell counts from five SEM images at 5000X magnification (n=100) by two independent researchers.

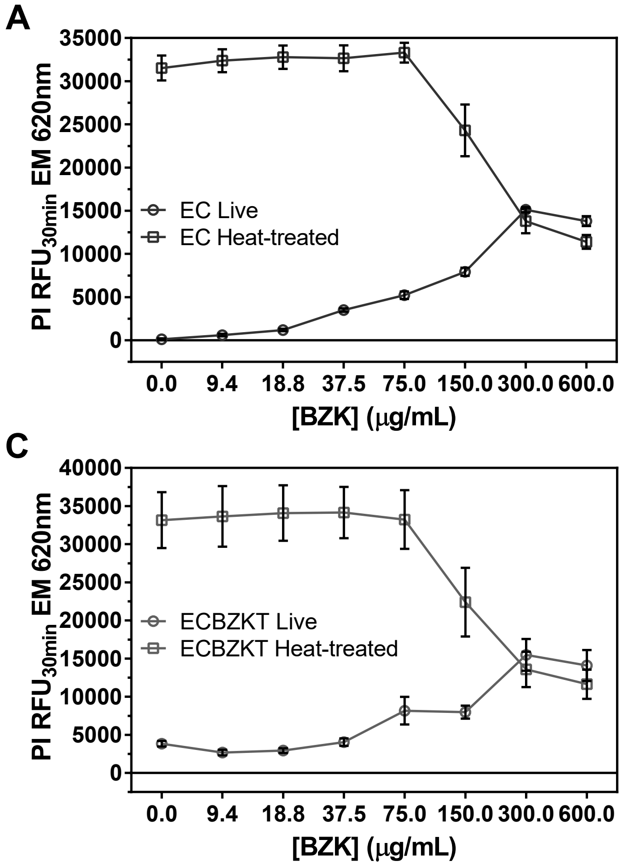

Figure S1. A comparison of RFDMIA heat-treated and live EC, ECCETT, and ECBZKT after 30-minute exposure to increasing concentrations of QAC. In all panels, RFDMIA PI RFU_{30min} at EM 620nm was measured. **A**) EC live (circle) and heat-treated cells (square) after 30-minute BZK exposure. **B**) EC live and heat-treated cells after 30-minute CET exposure. **C**) ECBZKT live (circle) and heat-treated (square) cells after 30-minute exposure to BZK. ECBZKT live cells at 0 µg/mL of BZK shows high PI RFU_{30min} EM 620nm values that diminish until 37.5 ug/mL BZK. **D**) ECCETT live (circle) and heat-treated (square) cells after 30-minute exposure to increasing concentrations of CET. ECCETT live cells at 0 µg/mL CET reveals high PI RFU_{30min} EM 620nm values that diminish until 37.5 µg/mL CET. Data shown is the mean RFU_{30min} values of 3 biological and 3 technical replicates. Error bars represent standard deviation within the samples.

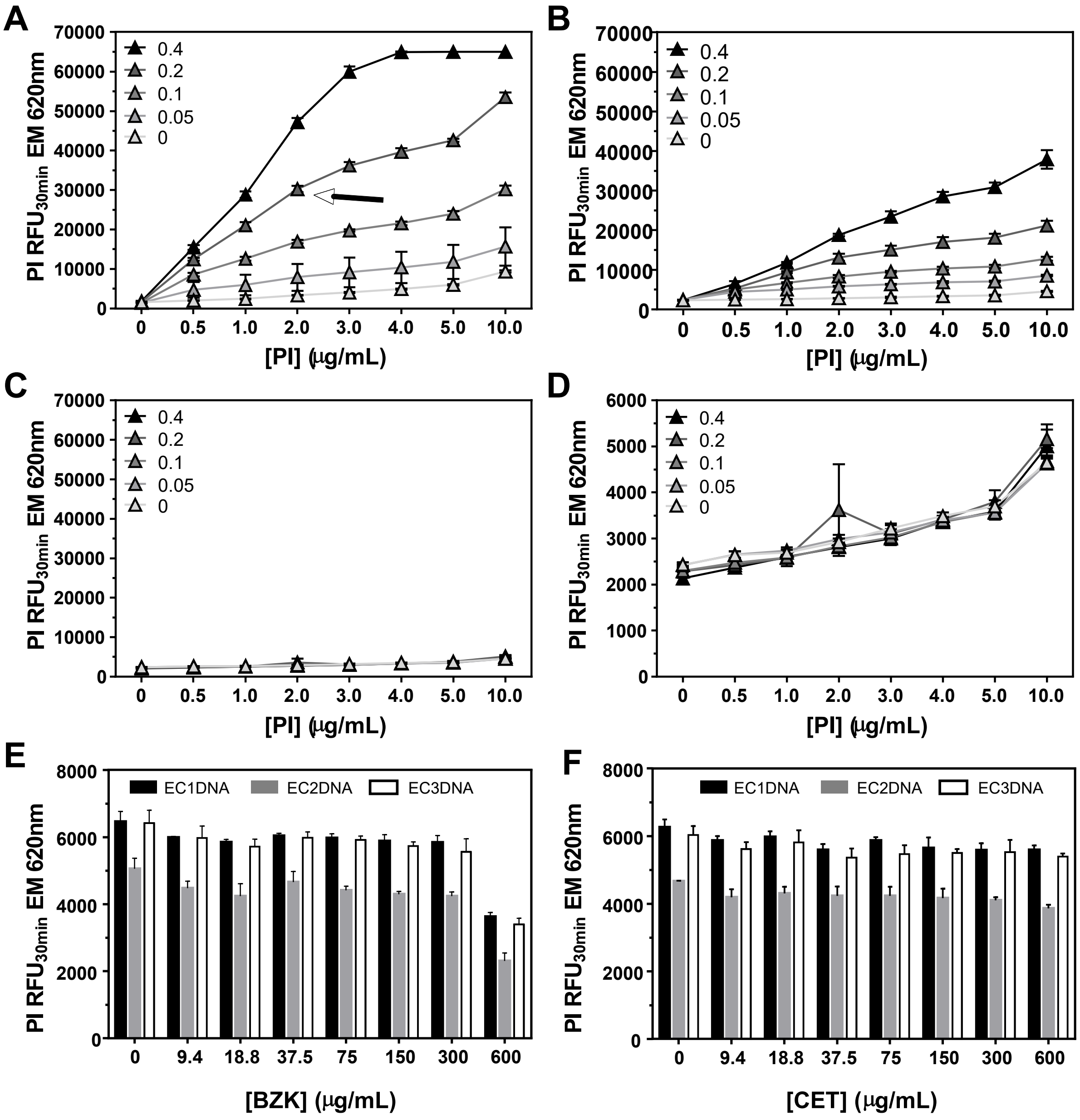

Figure S2. RFDMIA range finding experiments to determine optimal concentrations of PI with cells, DNA and various QACs. **A)** RFU of heat-treated EC cells at increasing OD_{600nm} **B)** RFU of DNA extracted from EC at increasing OD_{600nm} **C)** RFU of live cells at increasing OD_{600nm} **D)** RFU of live cells at increasing OD_{600nm} with a smaller EM range. Data represents 3 biological and 3 technical replicates with error bars indicating variation among the samples. The solid black arrow in panel A indicates the concentration of PI and bacterial cell OD_{600nm} to be utilized in all subsequent RFDMIAs. Range of OD_{600nm} is colored by grayscale gradient with OD_{600nm} 0 (Light grey), 0.05, 0.1, 0.2, 0.4 (Black) as shown in each figure panel legends. **E**) RFU of EC DNA at 2.0 µg/mL PI and increasing BZK concentrations in µg/mL. **F)** RFU of EC DNA at 2.0 µg/mL PI and increasing CET concentrations in µg/mL. In panels E and F, biological replicates of each EC DNA extraction (1-3) are shown and error bars show the standard deviation of y-axis values.

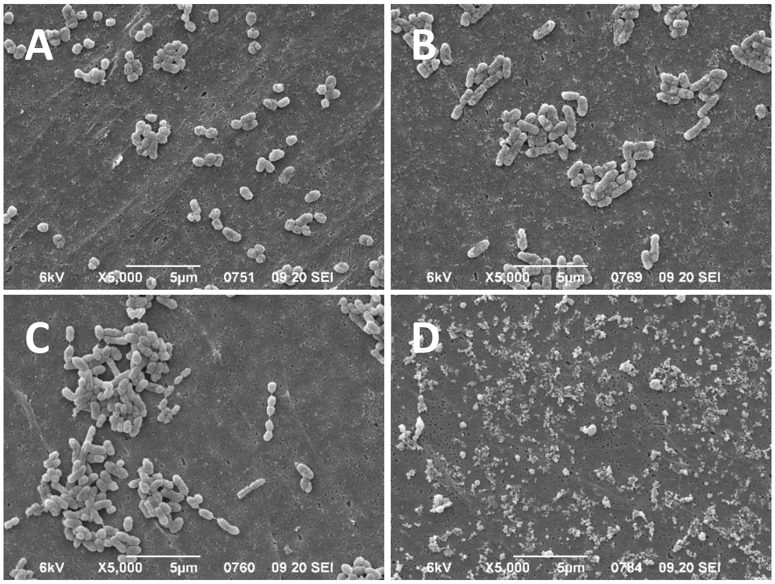

Figure S3. SEM images of *E coli* isolates EC (**A**), ECBZKT (**B**), ECCETT(**C**), and PBS only (**D**) controls after heat-treatment. All images are representative of five SEM images collected at 5000X magnifications and the white scale bar at the bottom of each panel image indicates 5 μm length.

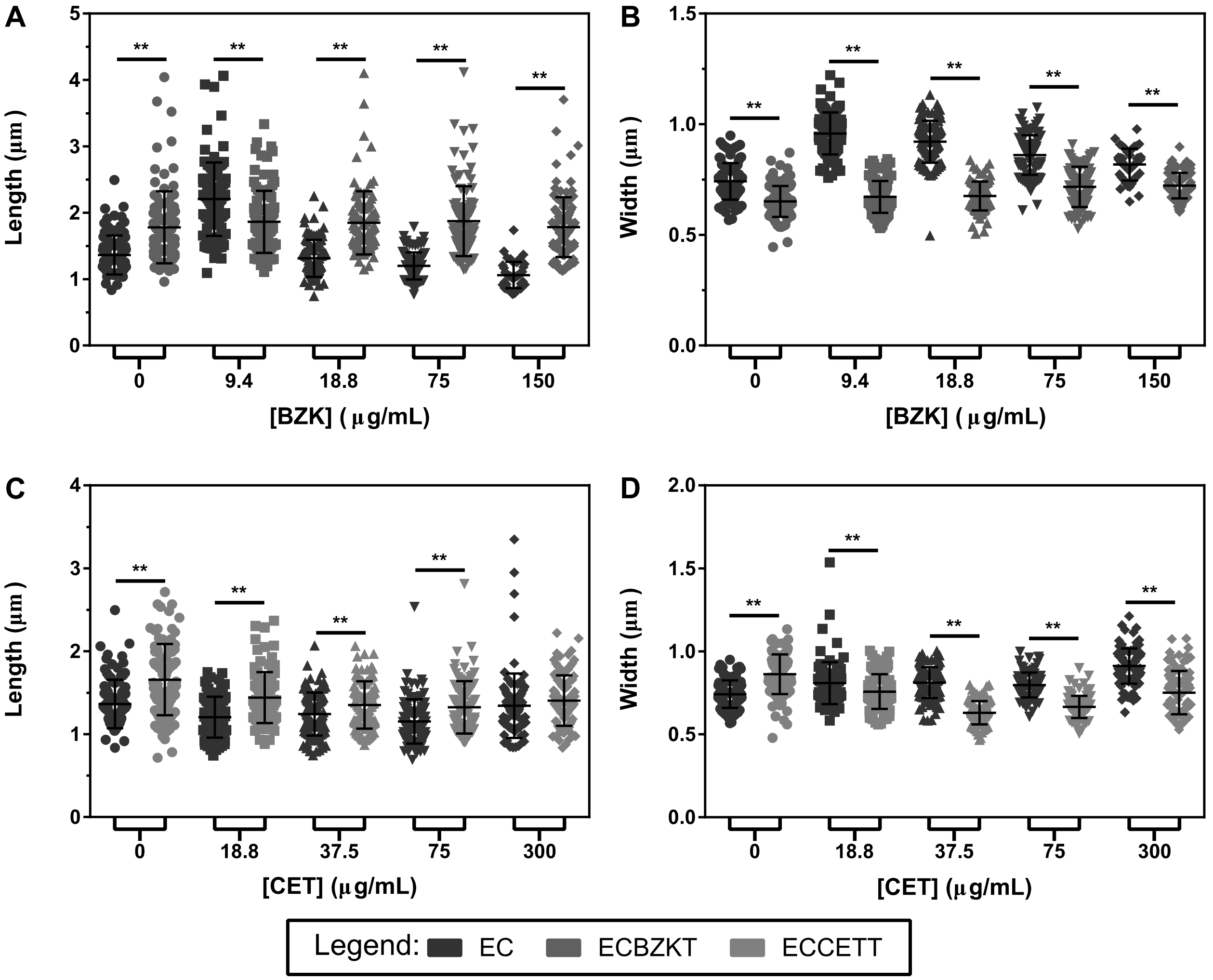

Figure S4. A summary of measure *E. coli* isolates' cell length and width measurements at various QAC concentrations from SEM images. Panels **A, C** show cell lengths and panels **B, D** show cell width measurements in μm using ImageJ[34] for isolates exposed to increasing concentrations of BZK (**A, B**) or CET (**C, D**). Asterisks (**) shown above bars in each panel indicate significantly different *P*-values (<0.01) between the samples; n=100. Differences in mean length and width are found in Table S1 (BZK) and S2 (CET). Error bars represent standard deviation from the central mean bar.

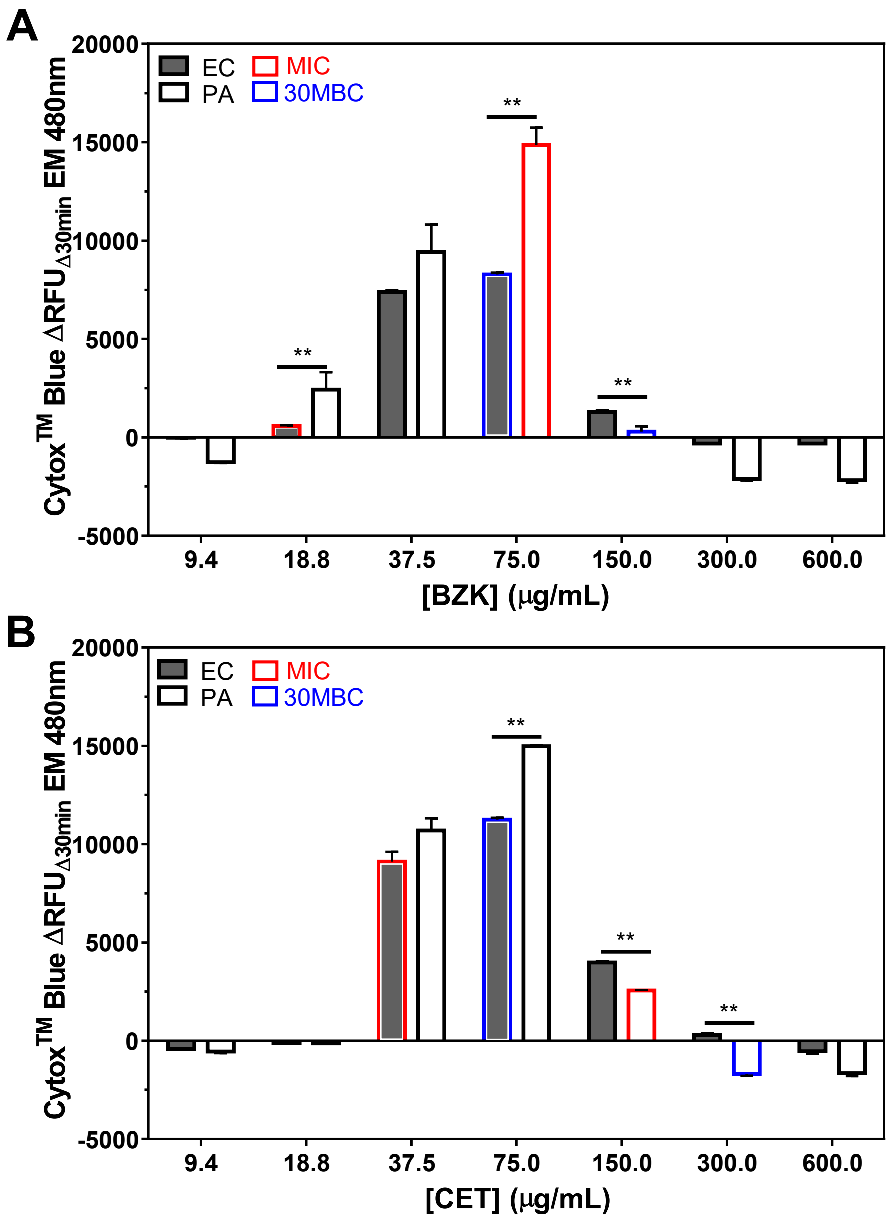

Figure S5. RFDMIA results using of Sytox™ Blue (EX/EM: 444nm/480 nm) as the impermeant fluorescent dye to detect the BZK susceptibility of *E. coli* (EC) and *P. aeruginosa* (PA). BZK (**A**) or CET (**B**) for *E. coli* (EC) and *P. aeruginosa* (PA). The RFDMIA was not able to predict the increased tolerance of PA. Students two-tailed t-tests were performed to detect significant differences in EM values between EC and PA at the same QAC concentration (**; *P*<0.01, n=3). Error bars represent standard deviation of three technical replicates. Red boxed values indicate QAC concentration MIC of the isolate. Blue boxed values represent the 30MBC QAC concentration.
